# The gut microbiome of Baka forager-horticulturalists from Cameroon is optimized for wild plant foods

**DOI:** 10.1016/j.isci.2024.109211

**Published:** 2024-02-10

**Authors:** Simone Rampelli, Sandrine Gallois, Federica D’Amico, Silvia Turroni, Marco Fabbrini, Daniel Scicchitano, Marco Candela, Amanda Henry

**Affiliations:** 1 Unit of Microbiome Science and Biotechnology, Department of Pharmacy and Biotechnology (FaBiT), Alma Mater Studiorum – University of Bologna, 40126 Bologna, Italy; 2 Department of Archaeological Sciences, Faculty of Archaeology, Leiden University, 2311 Leiden, the Netherlands; 3 Institute of Environmental Science and Technology, ST, 08193 Bellaterra, Spain; 4 Microbiomics Unit, Department of Medical and Surgical Sciences (DiMeC), Alma Mater Studiorum – University of Bologna, 40138 Bologna, Italy

**Keywords:** Microbiome, Diet

## Abstract

The human gut microbiome is losing biodiversity, due to the “microbiome modernization process” that occurs with urbanization. To keep track of it, here we applied shotgun metagenomics to the gut microbiome of the Baka, a group of forager-horticulturalists from Cameroon, who combine hunting and gathering with growing a few crops and working for neighboring Bantu-speaking farmers. We analyzed the gut microbiome of individuals with different access to and use of wild plant and processed foods, to explore the variation of their gut microbiome along the cline from hunter-gatherer to agricultural subsistence patterns. We found that 26 species-level genome bins from our cohort were pivotal for the degradation of the wild plant food substrates. These microbes include Old Friend species and are encoded for genes that are no longer present in industrialized gut microbiome. Our results highlight the potential relevance of these genes to human biology and health, in relation to lifestyle.

## Highlights

• Microbiome changes in relation to the consumption of wild plant foods
• Microbes from Baka encoded for genes no longer present in industrialized microbiome
• The consumption of wild plant foods is associated with 26 bacterial strains

## Graphical abstract

**Figure:**
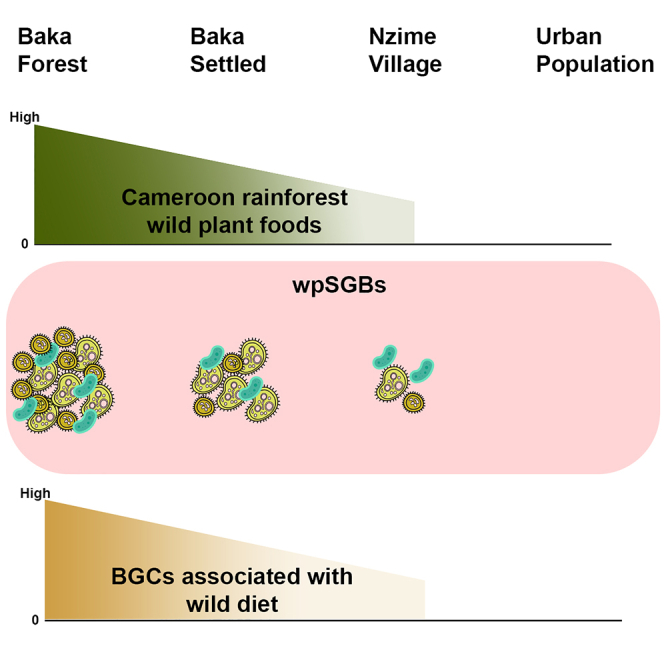

## Introduction

The human gut microbiome (GM) is capable of acquiring structural and functional configurations that reflect differences in modes of living. GM profiles vary considerably between groups that practice hunting and gathering and rural pastoralism and those that live in more industrialized and urban contexts.^1^^,^^2^^,^^3^^,^^4^^,^^5^^,^^6^^,^^7^^,^^8^^,^^9^^,^^10^^,^^11^^,^^12^^,^^13^^,^^14^ The former are characterized by a GM ecosystem with significant higher biodiversity, an extraordinarily complex glycome, and the presence of *Prevotella*, *Succinivibrio*, and *Treponema*, commonly referred to as "old friends" bacteria.^15^^,^^16^ In contrast, urban and industrialized groups more commonly display reduced ecosystem diversity, a complex resistome, and considerable number and complexity of genes specifically related to the metabolism of xenobiotic compounds. The differences between these two ends of the microbiome spectrum may provide glimpses of a possible adaptive GM response at the holobiont level, where the GM complements the limited plasticity of our genomes, providing the necessary phenotypic plasticity to adapt to the various lifestyles.^17^ For example, the increased structural and functional diversity typical of the GM from communities practicing gathering, small-scale horticulture, and pastoralism is likely a response to the diverse and refractive plant polysaccharides.^18^ In contrast, the GM from industrialized urban societies is more specialized for the metabolism of simple sugars and is more able to adapt to or detoxify the xenobiotic substances that they regularly encounter.^16^ However, numerous studies have also indicated that the GM among increasingly industrialized populations undergoes several deleterious changes, including a reduction in diversity and increase in functional specialization, that lead to reduced resilience, high risk of dysbiotic transitions, and increased burden of non-communicable diseases (e.g.,^19^). This rises the important concern of the “microbiome modernization process,"^16^ as a progressive and maladaptive shrinkage of the phylogenetic and functional diversity that is occurring along with the human urbanization and modernization processes.

By studying the diversity of the human GM globally and at the metapopulation level, we therefore gain insight on how these communities of bacteria, viruses, and fungi change in the human population, contributing to human health and our ability to succeed while engaging in a large diversity of lifeways or including, in some circumstances, possible maladaptive changes. Exploration into the GM profiles of non-urban industrialized groups is of particular importance, for two reasons. First, the full diversity of the human GM is still largely unexplored, with a limited knowledge of its variation among rural and traditional population, which may still represent an untapped source of probiotic functions loss in urbanized context. Second, sociopolitical pressures have meant that many groups practicing foraging, horticulture, and pastoralist lifestyles are increasingly adopting aspects of the urban industrial lifestyle, including consumption of mass-produced foods, regular use of antibiotics, and greater reliance on a smaller number and variety of food items.^8^^,^^12^ There is therefore an increased urgency to capture information about the unique GM configurations across as wide a spread of different lifeways, in order to highlight and protect their strategic functional traits providing selected and important phenotypes. Furthermore, by exploring the GM configurations of groups who rely heavily on the consumption of diverse plants, we may highlight the importance of a more sustainable plant-based diet in industrialized urban populations, which would result not only in the recovery of the strains/genes necessary for the exploitation of complex plant polysaccharides but also in the concomitant gain of associated probiotic functions and/or metabolites, with important benefit in terms of human health.

In this context, we partnered with the Baka, a group of forager-horticulturalists from southeastern Cameroon, who combine hunting and gathering with growing a small number of crops and working for the neighboring Bantu-speaking farmers (the Nzime).^20^ Part of the food consumed by the Baka, particularly cassava (*Manihot esculenta*) and plantain (*Musa paradisiaca*), comes from agricultural fields,^21^ with the addition of only few processed foods (i.e., stock cubes, tomato sauce, and, rarely, sardines). On the other hand, a wide variety of key nutrients come from game (meat) and forest foods, including edible wild plants and nuts.^22^ We characterized the GM of Baka who spent a large amount of their time in a forest camp (Baka forest group), Baka individuals who mostly live in a village along the logging road (Baka settled group), and Nzime farmers (Nzime village group). The Baka forest group consumed more wild plants and less processed foods than the settled Baka, whereas the Nzime village group relied more on processed and traded foods. Recruiting a cohort with individuals who showed different accessibility to wild plant foods and processed foods, we have been able to highlight the GM features associated with the specificities of the three rural lifestyles, with Baka forest group relying the most on western African rainforest wild foods. Metagenomic profiles from this cohort were interpreted across subsistence strategies and integrated with available data from worldwide populations, with varying degrees of traditional or industrialized lifestyles. We explored the variation of the GM along the transition from hunter-gatherer to agricultural communities at a finer functional resolution than previous efforts, from the microbiome network topology to the genome scale metabolic models, until species-level genome bins. Our work led to uncovering specific adaptive gradients associated with the consumption of western African rainforest wild plant foods, at both taxonomic and functional scale. Finally, the results herein offer a complete description of reference genomes of microbes associated with wild plant foods consumption and their potential relevance for human health.

## Results

### Baka, Nzime, and lifestyle gradient

**Figure 1:**
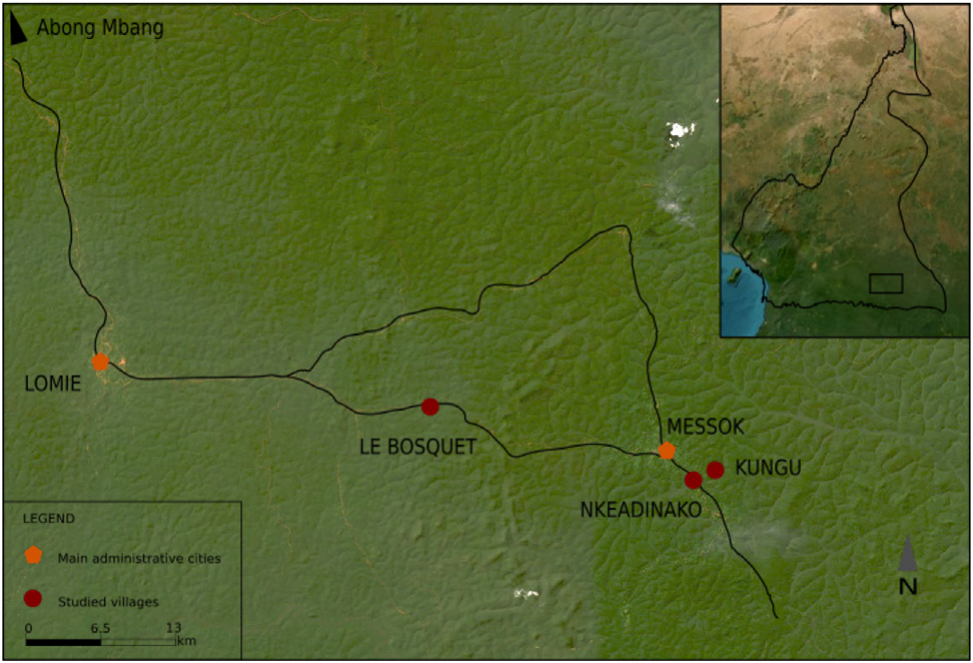
Geographic map of sample collection locations Baka and Nzime live in South-East part of Cameroon, as indicated in the box on the top-right of the figure. Fecal samples from Baka adults were collected in the Kungu forest camp (Baka forest) and in the Le Bosquet village (Baka settlement). Samples from Nzime individuals were collected in the Nkeadinako village. Such locations are indicated by red dot on the map.

This study was part of a larger project exploring foraging strategies and the use of western African rainforest wild plants among the Baka people of southeastern Cameroon.^20^^,^^22^^,^^23^^,^^24^ In this study, we asked for volunteers from three different communities, representing the two major ethnic groups in the region, the Baka and the Nzime. The Baka are Ubangian-speaking people who practice foraging and small-scale cultivation, whereas the Nzime are Bantu-speaking people who practice subsistence-level agriculture, including, to a reduced extent, animal husbandry. Both groups live in the same area, primarily in villages clustered along logging roads (Figure 1). The two groups regularly interact with the Baka trading forest foods (plants and game) for agricultural crops grown by the Nzime. The Baka also engage in wage labor for the Nzime in their agricultural fields, for logging companies and collect forest products for sale to traders along the logging roads. Small market stalls that sell canned goods, candy, alcohol, and other items also are present along the logging roads in the Nzime villages, and the Baka have some access to these resources.^20^ Although many Baka have homes and fields in one of the villages, some regularly live in forest camps that are located several hours’ walk from the logging roads. These forest camps are sometimes used only seasonally (e.g., during the rainy seasons when forest nuts are collected), but some individuals choose to spend the majority of their time in the forest as well.

In this study, we compared the GM profiles from 26 Baka participants, including 16 individuals from the forest camp (Baka forest) and 10 individuals from one of the settled villages (Baka settlement, see Figure 1). Additionally, 18 individuals were recruited from the neighboring Nzime (Nzime village).

### Baka and western African rainforest wild plant foods

The Baka use a variety of wild plant foods in their meals: (1) dark green leaves (e.g., *Gnetum africanum*, Baka name: *koko*), which are rich in amino acids and are likely an important source of protein; (2) oil from nuts, particularly *Irvingia* spp (bush mango, Baka name: *payo*, *pekoe*), *Baillonella toxisperma* (Baka name: *màbè*) and *Panda oleosa* (Baka name: *kanà*) that are rich in fat and used for cooking; and (3) spices such as *Afrostyrax lepidophyllus* (Baka name: *ngimbà*) and a variety of fruits, mushrooms, bark, and other plant species. Evidence from our previous research in this community^21^ outlined that Baka villagers settled closest to the market town consumed legumes, nuts, and seed, but not specifically coming from the wild. On the other hand, Baka who spent more time in the forest have more access to the wild plant foods and consumed them with more regularity and at higher quantity. Finally, individuals from the Nzime village possess more money, which is reflected in wider access to other food and less interest toward wild plant food consumption.

### Baka GM varies on the basis of lifestyle

**Figure 2:**
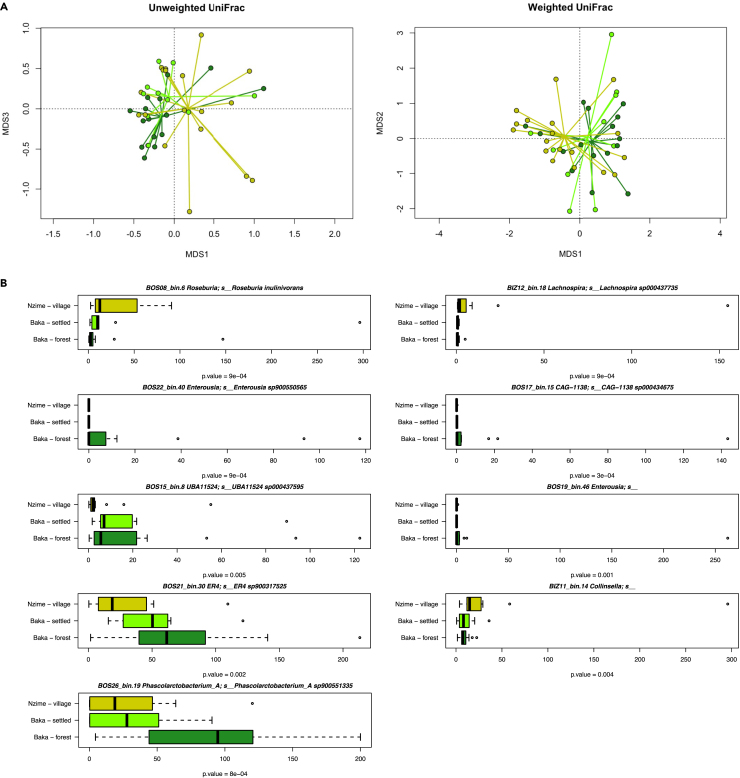
Differences in GM compositions among individuals of the Baka forest (green), Baka settled (light green), and Nzime village (yellow-green) groups (A) PCoA plots based on unweighted and weighted UniFrac distances and (B) boxplots for SGBs abundances, in terms of genome copies per million of sequenced reads. p values are obtained using Kruskal-Wallis test. See also Figure S2 for the distribution of all the SGBs across the entire cohort and Figure S3 for the same analysis combining Baka forest and Baka settled in a unique group.

In order to assess whether the GM varies across lifestyle, we characterized the samples by shotgun metagenomics, with an average of 8.1 M (±1.9 M) high-quality reads per samples (Table S1). Starting from the 44 metagenomes, we were able to reconstruct 628 metagenomes-assembled genomes (MAGs), which were dereplicated to 161 species-level genome bins (SGBs, i.e., clusters of MAGs spanning 5% genetic diversity; see the “STAR Methods” section and the Figure S1 for further details). Then, we mapped such 161 representative genomes against the previously available SGBs database from Pasolli et al.*,*^25^ that described the >1,50,000 MAGs from the GM of different individuals, spanning age, geography, and lifestyle. In total, 132 of our SGBs (82%) cluster together with at least one known SGB from Pasolli et al.^25^ (Table S2); on the other hand, the remaining fraction of SGBs (29 SGBs, 18%) showed >5% genetic distance to any SGBs of the available database, representing candidate new taxa. Based on the 161 SGBs, comparison of community structure in the three groups (Baka forest, Baka settled, and Nzime village), using weighted and unweighted UniFrac distances, showed that the GM varies across groups (p = 0.001, permutational test with pseudo-F ratio). In particular, visualization of these distances using principal coordinates analysis (PCoA) revealed separation between the Baka forest and Nzime village individuals (p value = 0.001, permutation test with pseudo-F ratios), with the Baka settled group closely associated with the Baka forest group, but slightly shifted toward the Nzime village group, as reflecting the changes in lifestyle (Figure 2A).

Coherently, several SGBs mirror this trend, with SGBs for *Roseburia inulinivorans*, *Collinsella*, and *Lachnospira sp000437735* that significantly decreased from Bantu-speaking individuals to Baka forest, with Baka settled showing an intermediate abundance, and on the other hand, SGBs for *Enterousia*, *unknown Ruminococcaceae*, and *Phascolarctobacterium sp90055135*, showing the opposite trend (Figure 2B, p < 0.05, Kruskall-Wallis rank-sum test; see on the figure panel for the exact p values). When combining the two Baka groups into one and compare it with the Nzime, all the differences mentioned earlier were generally confirmed, with the only exception for the two SGBs assigned to *Enterousia*, which were characteristic only of the Baka Forest group. However, additional differences have been also observed, such as *Agathobacter rectalis*, significantly higher in the Nzime, and *Faecousia* and *unknown Ruminococcaceae*, characterizing the Baka (Figure S3).

### Identification of SGBs involved with western African rainforest wild plant food consumption and their contribution to GM structure

To capture the functional diversity of GM, encoding for the metabolic functions able to use wild plant foods as substrate, we applied a *de novo* functional screening of genome-scale metabolic networks (GSMNs) to the full set of SGBs characterized within this study. In particular, we used Metage2Metabo (M2M),^26^ a resource that allows the identification of the key microbiome components for specific substrate usage, with a particular emphasis on metabolic cooperation. Based on the frequency of wild plant species appearing in the dietary recalls of our previous work (N = 2377),^22^ we ran M2M for the five wild plant foods that showed a frequency intake >1%, which included *Gnetum africanum* (40.7%), *Irvingia* spp (8.4%), *Baillonella toxisperma* (5.9%), *Afrostyrax lepidophyllus* (1.5%), and *Panda oleosa* (1.3%).

**Figure 3:**
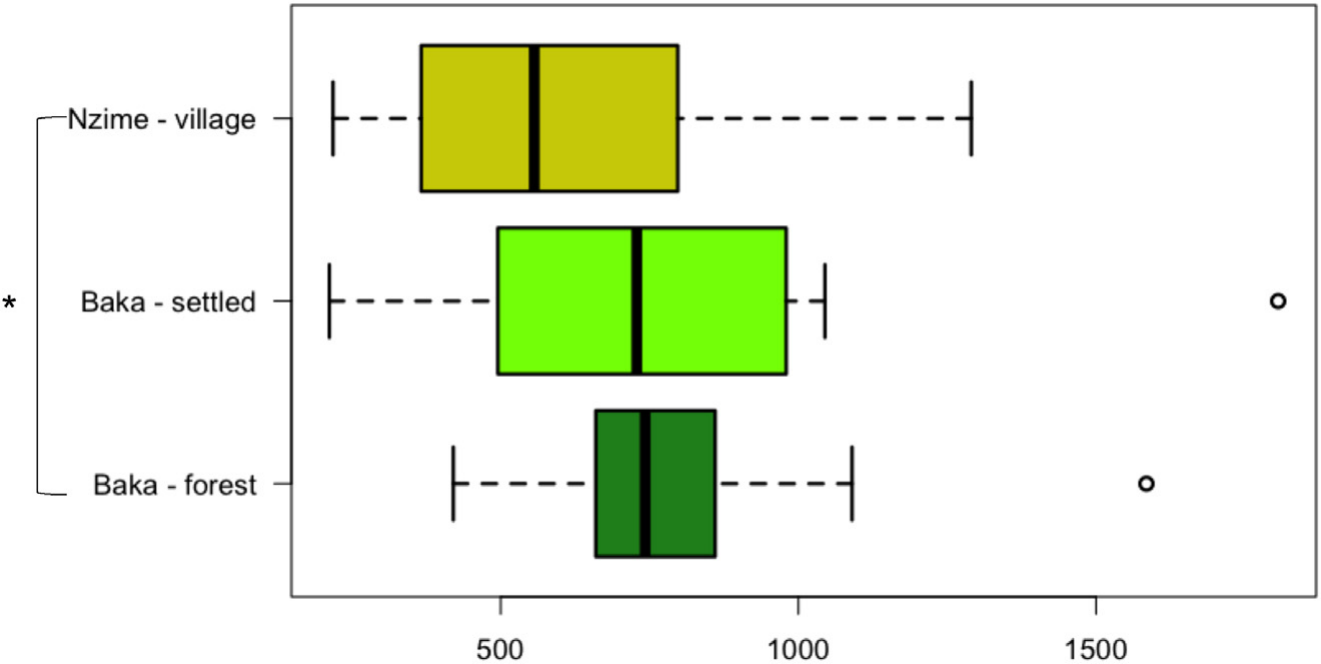
Comparison of cumulative abundances of wpSGBs Highlighting comparison between fecal samples from individuals of the Baka forest (green), Baka settled (light green), and Nzime village (yellow-green) groups, represented by boxplots. Values in genome copies per million reads. ∗p = 0.02, Kruskal-Wallis rank-sum test.

We found a module of 26 cooperating SGBs, out of 161, as essential for the metabolism of the western African rainforest wild plant food substrates (wpSGBs). In particular, these wpSGBs included taxa that are usually associated with a rural lifestyle, such as *Treponema*, *Succinivibrio*, and *Prevotella*, together with other taxa that are common components of GM also in industrialized context, such as *Butyricicoccus*, *Dialister*, *Escherichia coli*, *Phascolarctobacterium*, and *Ruminococcus*. Notably, *Treponema*, *Succinivibrio*, and *Prevotella* are Old Friend species, usually considered part of the human GM in our ancestors before adopting agriculture,^27^ and often absent in “Western” populations.^18^ Straightly—and supporting the connection between the wpSGBs module and the African rainforest wild food consumption—the cumulative abundance of wpSGBs was significantly higher in the Baka forest group, compared with individuals of the Nzime village (p = 0.02, Wilcoxon rank-sum test), with the Baka settled group showing an intermediate abundance (Figure 3).

24 wpSGBs showed a representative (kSGBs) in the SGBs database from Pasolli et al.^25^ However, most of them (23/24) are still rather uncharacterized species, as they represent sequenced genomes assigned to genus-, family-, or order-level without any species name. Many such unknown wpSGBs were from the Clostridia class (10 kSGBs). Further, the 2 remaining wpSGBs that fall within previously uncharacterized genomes (i.e., those showed >5% genetic distance to any SGBs of the database) were assigned to *Collinsella* and to an uncharacterized species of the class Bradymonadia. A full list of the wpSGBs and their taxonomic assignment is available in Table S3.

**Figure 4:**
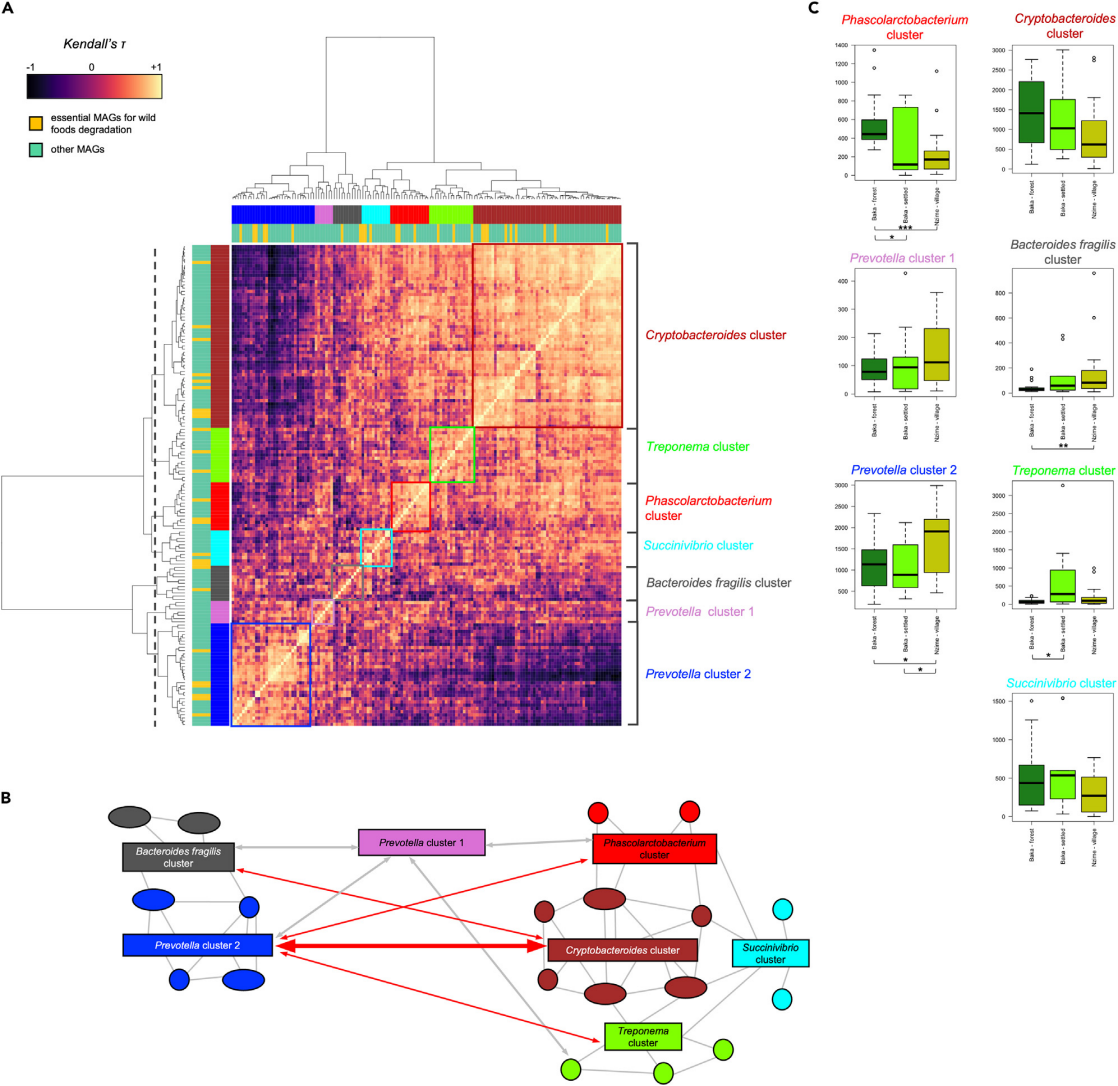
Co-abundance analysis highlights distinct bacterial networks characterizing the three groups (A) A network heatmap based on Kendall’s correlation coefficient and GM data was generated using the most abundant SGBs across all samples (see complete list of taxa and their abundance in Table S4). The most dominant clusters identified are highlighted by different colored boxes and were confirmed by permutation tests with pseudo-F ratios (p < 0.05, adonis of the R package vegan). One setting was used for cluster analysis (gray dashed lines), which identified seven clusters. The *Cryptobacteroides* cluster is highlighted in brown, the *Treponema* cluster in green, the *Succinivibrio* cluster in cyan, *Phascolarctobacterium* in red, *Prevotella* (cluster 1) in pink, *Bacteroides fragilis* in gray, and *Prevotella* (cluster 2) in blue. (B) Network scheme illustrating the relationships between bacterial clusters. The leading taxa in each network are highlighted. A positive correlation is shown with a gray line and a negative correlation with a red line. Clusters are colored as in (A). (C) Cumulative relative abundance of the different clusters of taxa among the three groups (∗p < 0.01, ∗∗p < 0.001; Wilcoxon test). See also Figure S4.

We next investigated how these 26 wpSGBs contributed to the overall GM structure and community topology in our dataset. To this purpose, we constructed a heatmap based on the Kendall’s tau correlation coefficients between the different 150 SGBs with a minimum genome copies per million reads of 10 in at least two samples. We then grouped correlated bacterial species into seven clusters of SGBs, represented by different colors, whose interactions are represented by Wiggum plot, where SGBs abundance is proportional to the circle diameter (Figures 4A, 4B, and S4, and Table S4). The dominant SGBs for each cluster were taxonomically assigned to *Bacteroides fragilis* (gray), *Cryptobacteroides* (brown), *Prevotella* (pink, *Prevotella* cluster 1), *Prevotella* (blue, *Prevotella* cluster 2), *Phascolarctobacterium* (red), *Succinivibrio* (cyan), and *Treponema* (green). The topological data analysis indicated that *Cryptobacteroides*, *Prevotella* (from cluster 2), and *Treponema* are keystone taxa in the GM network structure, showing the highest connectivity, due to the combination of high values of (1) closeness centrality (0.50, 0.55, and 0.46, respectively), (2) betweenness centrality (0.03, 0.02, and 0.04, respectively), and (3) degree (29, 48, and 20 respectively), with a normalized genome counts per million reads >50. Notably, two wpSGBs were assigned to two of these keystone taxa (*Treponema* and *Prevotella*).

The clusters changed in abundance across the three groups, with the 26 wpSGBs associated with wild plant food consumption that showed a higher representation in the *Cryptobacteroides* cluster (n. wpSGBs = 9), with respect to *Prevotella* cluster 2 (6), *Phascolarctobacterium* (3), *Treponema* (3), *Succinivibrio* (3), and *Bacteroides fragilis* (2) clusters (Figures 4C and S4, and Table S4). In particular, the GM of the Baka forest group was characterized by a Cryptobacteroides-centered cluster, with relevant contribution in terms of abundance of the *Phascolarctobacterium*, *Treponema*, and *Succinivibrio* clusters. Conversely, the GM of the Nzime group was found to be centered around the *Prevotella* cluster 2 and *Bacteroides fragilis* cluster.

As expected, the GM of the Baka settled group was characterized by an intermediate configuration between the Baka forest and the Nzime village groups, coherently with their lifestyle that represented an intermediate between the other two groups. Indeed, we observed a strong resilience of members of the *Cryptobacteroides* cluster, to values comparable with the Baka forest, together with the emergence of some members of the *Prevotella* cluster 2. Notably, this group was also characterized by a higher abundance of members of the *Treponema* cluster than the other two groups.

Collectively, the different accessibility to wild plant foods was probably the reason for the modifications of GM structure in Baka individuals, as revealed in the Baka settled group with respect to Baka forest, with new emerging GM traits that are shared with Nzime agriculturists (Nzime village group). In order to rule out possible strain transfer between individuals from the Baka settled and Nzime village groups, we applied StrainPhlAn3^28^ to the most abundant wpSGBs. We found that no strains were shared, as if wpSGBs from different groups were different strains or, at least, not deriving from recent transmission events between individuals.

Although the abundance of the wpSGB module decreased when comparing Baka forest and Baka settled groups, wpSGBs were preserved in almost all clusters and samples, including the Nzime village group, even if at lower abundance. This persistence likely had two contributing causes: (1) western African rainforest wild plant food consumption only decreases across these three groups, but never completely ceased, and (2) the metabolic capabilities of wpSGBs were very varied and not exclusively limited to degradation of wild-plant-food-deriving substrates, but possibly also providing additional probiotic functions of relevance for keeping health.

### Wild-plant-food-associated taxa contain genes that are not present in the industrialized GM

**Figure 5:**
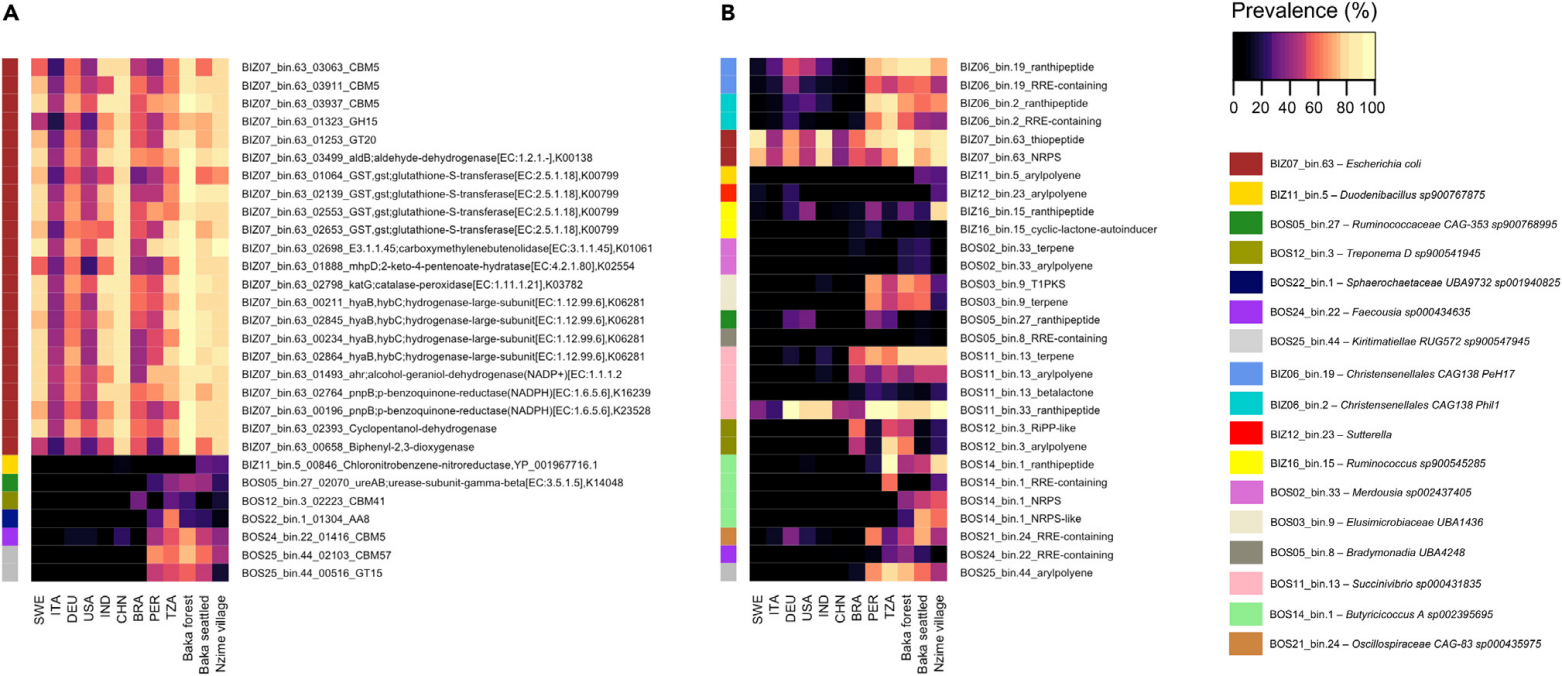
Prevalence of wpSGB genes and BGCs across human populations Heatmaps showing the prevalence of the 29 wpSGB genes showing a propensity for the degradation of polysaccharides and phytochemicals (A) and of 34 BGCs for secondary metabolites (B), which were not present in the other SGBs. Datasets comprised individuals relying on both rural and industrialized lifestyle from different geographical origin (see also Table S5 for further details). SWE, Sweden; ITA, Italy (industrial); DEU, Germany (industrial); USA, USA (industrial); IND, India (industrial); CHN, China (industrial); BRA, Brasil (rural); PER, Peru (rural); TZA, Tanzania (rural).

In order to search for the specific wpSGBs functional features that associated with the metabolism of wild plant food substrates (i.e., genes involved in degradation of the molecules contained within *Gnetum africanum*, *Irvingia* sp., *Baillonella toxisperma*, *Afrostyrax lepidophyllus*, and *Panda oleosa*), we first screened wpSGBs for genes for the degradation of polysaccharides and phytochemicals (e.g., polyphenols and essential oils) and that were not present in the remaining 135 SGBs from this study (see STAR Methods for more details). We found 29 genes from 7 different wpSGBs with these characteristics (Table 1). In particular, the full list contains 22 genes from *E. coli*, together with seven genes belonging to six different wpSGBs, encoding for urease (wpSGB taxonomy: unclassified Clostridia), chloronitrobenzene-nitroreductase (*Duodenibacillus*), pullulanase (*Treponema*), dihydrolipoyl dehydrogenase (unclassified Sphaerochaetaceae), sialidase (*Faecousia*), mannosyltransferase, and a protein assigned to the CBM57 module (unclassified Kiritimatiellae). We then verified the presence of such genes within the gut metagenomes of 970 individuals of different geographical origin that relied on rural or industrial lifestyle (Figure 5A; Table S5). The distribution of these functional features in the human gut metagenomes suggests, from one hand, that the *E. coli*-related genes are widespread, and, on the other side, that the remaining functions are most likely exclusive of rural GM, irrespectively of geographic origin, with the exception of the chloronitrobenzene-nitroreductase, which is exclusively present in our cohort.

**Table 1:** List of the 29 wpSGB genes showing a propensity for the degradation of polysaccharides and phytochemicals that were not present in the other SGBs

wpSGBs	Taxonomy	Length	Prokka assignment to dbCAN and Xenopath databases	BLASTP against nr NCBI
BIZ07_bin.63	d__Bacteria; p__Proteobacteria; c__Gammaproteobacteria; o__Enterobacterales; f__Enterobacteriaceae; g__Escherichia; s__Escherichia coli	2694	CBM5	Bifunctional chitinase/lysozyme [Escherichia coli]
BIZ07_bin.63	d__Bacteria; p__Proteobacteria; c__Gammaproteobacteria; o__Enterobacterales; f__Enterobacteriaceae; g__Escherichia; s__Escherichia coli	450	CBM5	Chain A, Potassium-binding protein Kbp [Escherichia coli K-12]
BIZ07_bin.63	d__Bacteria; p__Proteobacteria; c__Gammaproteobacteria; o__Enterobacterales; f__Enterobacteriaceae; g__Escherichia; s__Escherichia coli	1221	CBM5	murein transglycosylase D [Escherichia coli]
BIZ07_bin.63	d__Bacteria; p__Proteobacteria; c__Gammaproteobacteria; o__Enterobacterales; f__Enterobacteriaceae; g__Escherichia; s__Escherichia coli	1611	GH15	Glycoside hydrolase family 15 protein [Enterobacteriaceae]
BIZ07_bin.63	d__Bacteria; p__Proteobacteria; c__Gammaproteobacteria; o__Enterobacterales; f__Enterobacteriaceae; g__Escherichia; s__Escherichia coli	1425	GT20	Alpha, alpha-trehalose-phosphate synthase [Escherichia coli]
BIZ07_bin.63	d__Bacteria; p__Proteobacteria; c__Gammaproteobacteria; o__Enterobacterales; f__Enterobacteriaceae; g__Escherichia; s__Escherichia coli	1026	aldB; aldehyde-dehydrogenase[EC:1.2.1.-],K00138	L-Threonine 3-dehydrogenase [Escherichia coli]
BIZ07_bin.63	d__Bacteria; p__Proteobacteria; c__Gammaproteobacteria; o__Enterobacterales; f__Enterobacteriaceae; g__Escherichia; s__Escherichia coli	411	GST, gst; glutathione-S-transferase[EC:2.5.1.18],K00799	1,4-Dihydroxy-2-naphthoyl-CoA hydrolase [Escherichia coli]
BIZ07_bin.63	d__Bacteria; p__Proteobacteria; c__Gammaproteobacteria; o__Enterobacterales; f__Enterobacteriaceae; g__Escherichia; s__Escherichia coli	1719	GST, gst; glutathione-S-transferase[EC:2.5.1.18],K00799	Ubiquinone-dependent pyruvate dehydrogenase [Escherichia coli]
BIZ07_bin.63	d__Bacteria; p__Proteobacteria; c__Gammaproteobacteria; o__Enterobacterales; f__Enterobacteriaceae; g__Escherichia; s__Escherichia coli	720	GST, gst; glutathione-S-transferase[EC:2.5.1.18],K00799	Purine-nucleoside phosphorylase [Escherichia coli]
BIZ07_bin.63	d__Bacteria; p__Proteobacteria; c__Gammaproteobacteria; o__Enterobacterales; f__Enterobacteriaceae; g__Escherichia; s__Escherichia coli	645	GST, gst; glutathione-S-transferase[EC:2.5.1.18],K00799	Glutathione transferase [Escherichia coli]
BIZ07_bin.63	d__Bacteria; p__Proteobacteria; c__Gammaproteobacteria; o__Enterobacterales; f__Enterobacteriaceae; g__Escherichia; s__Escherichia coli	1410	E3.1.1.45; carboxymethylenebutenolidase[EC:3.1.1.45],K01061	Glutamate--ammonia ligase [Escherichia coli]
BIZ07_bin.63	d__Bacteria; p__Proteobacteria; c__Gammaproteobacteria; o__Enterobacterales; f__Enterobacteriaceae; g__Escherichia; s__Escherichia coli	867	mhpD; 2-keto-4-pentenoate-hydratase[EC:4.2.1.80],K02554	2-Hydroxy-6-oxonona-2,4-dienedioate hydrolase [Escherichia coli]
BIZ07_bin.63	d__Bacteria; p__Proteobacteria; c__Gammaproteobacteria; o__Enterobacterales; f__Enterobacteriaceae; g__Escherichia; s__Escherichia coli	2298	katG; catalase-peroxidase[EC:1.11.1.21],K03782	Formate C-acetyltransferase [Escherichia coli]
BIZ07_bin.63	d__Bacteria; p__Proteobacteria; c__Gammaproteobacteria; o__Enterobacterales; f__Enterobacteriaceae; g__Escherichia; s__Escherichia coli	2547	hyaB, hybC; hydrogenase-large-subunit[EC:1.12.99.6],K06281	Trimethylamine-N-oxide reductase TorA [Escherichia coli]
BIZ07_bin.63	d__Bacteria; p__Proteobacteria; c__Gammaproteobacteria; o__Enterobacterales; f__Enterobacteriaceae; g__Escherichia; s__Escherichia coli	1689	hyaB, hybC; hydrogenase-large-subunit[EC:1.12.99.6],K06281	Fatty acyl-AMP ligase [Escherichia coli]
BIZ07_bin.63	d__Bacteria; p__Proteobacteria; c__Gammaproteobacteria; o__Enterobacterales; f__Enterobacteriaceae; g__Escherichia; s__Escherichia coli	1794	hyaB, hybC; hydrogenase-large-subunit[EC:1.12.99.6],K06281	Ni/Fe-hydrogenase large subunit [Escherichia coli]
BIZ07_bin.63	d__Bacteria; p__Proteobacteria; c__Gammaproteobacteria; o__Enterobacterales; f__Enterobacteriaceae; g__Escherichia; s__Escherichia coli	1704	hyaB, hybC; hydrogenase-large-subunit[EC:1.12.99.6],K06281	Hydrogenase 2 large subunit [Escherichia coli]
BIZ07_bin.63	d__Bacteria; p__Proteobacteria; c__Gammaproteobacteria; o__Enterobacterales; f__Enterobacteriaceae; g__Escherichia; s__Escherichia coli	735	ahr; alcohol-geraniol-dehydrogenase(NADP+)[EC:1.1.1.2	3-Oxoacyl-ACP reductase FabG [Escherichia coli]
BIZ07_bin.63	d__Bacteria; p__Proteobacteria; c__Gammaproteobacteria; o__Enterobacterales; f__Enterobacteriaceae; g__Escherichia; s__Escherichia coli	762	pnpB; p-benzoquinone-reductase(NADPH)[EC:1.6.5.6],K16239	Uridine phosphorylase [Escherichia coli]
BIZ07_bin.63	d__Bacteria; p__Proteobacteria; c__Gammaproteobacteria; o__Enterobacterales; f__Enterobacteriaceae; g__Escherichia; s__Escherichia coli	801	pnpB; p-benzoquinone-reductase (NADPH)[EC:1.6.5.6],K23528	Pyrimidine utilization protein D [Escherichia coli]
BIZ07_bin.63	d__Bacteria; p__Proteobacteria; c__Gammaproteobacteria; o__Enterobacterales; f__Enterobacteriaceae; g__Escherichia; s__Escherichia coli	984	Cyclopentanol-dehydrogenase	Quinone oxidoreductase [Escherichia coli]
BIZ07_bin.63	d__Bacteria; p__Proteobacteria; c__Gammaproteobacteria; o__Enterobacterales; f__Enterobacteriaceae; g__Escherichia; s__Escherichia coli	519	Biphenyl-2,3-dioxygenase	3-Phenylpropionate/cinnamic acid dioxygenase subunit beta [Escherichia coli]
BIZ11_bin.5	d__Bacteria; p__Proteobacteria; c__Gammaproteobacteria; o__Burkholderiales; f__Burkholderiaceae; g__Duodenibacillus; s__Duodenibacillus sp900767875	756	Chloronitrobenzene-nitroreductase, YP001967716.1	3-Oxoacyl-ACP reductase FabG [Duodenibacillus massiliensis]
BOS05_bin.27	d__Bacteria; p__Firmicutes_A; c__Clostridia; o__Oscillospirales; f__Ruminococcaceae; g__CAG-353; s__CAG-353 sp900768995	1203	ureAB; urease-subunit-gamma-beta[EC:3.5.1.5],K14048	Urease subunit alpha [Oscillospiraceae bacterium]
BOS12_bin.3	d__Bacteria; p__Spirochaetota; c__Spirochaetia; o__Treponematales; f__Treponemataceae; g__Treponema_D; s__Treponema_D sp900541945	666	CBM41	Type I pullulanase [Treponema socranskii]
BOS22_bin.1	d__Bacteria; p__Spirochaetota; c__Spirochaetia; o__Sphaerochaetales; f__Sphaerochaetaceae; g__UBA9732; s__UBA9732 sp001940825	1344	AA8	Dihydrolipoyl dehydrogenase [Spirochaetales bacterium]
BOS24_bin.22	d__Bacteria; p__Firmicutes_A; c__Clostridia; o__Oscillospirales; f__Oscillospiraceae; g__Faecousia; s__Faecousia sp000434635	2202	CBM5	Exo-alpha-sialidase [Anaeromassilibacillus senegalensis]
BOS25_bin.44	d__Bacteria; p__Verrucomicrobiota; c__Kiritimatiellae; o__RFP12; f__UBA1067; g__RUG572; s__RUG572 sp900547945	5061	CBM57	Autotransporter-associated beta strand repeat-containing protein [Kiritimatiellae bacterium]
BOS25_bin.44	d__Bacteria; p__Verrucomicrobiota; c__Kiritimatiellae; o__RFP12; f__UBA1067; g__RUG572; s__RUG572 sp900547945	849	GT15	Glycolipid 2-alpha-mannosyltransferase [uncultured archaeon]

Further, we hypothesized that some wpSGBs could encode for additional features not connected to degradation of plant substrates but relevant microbiome-microbiome and microbiome-host communication, with unexplored impact on human health.^29^^,^^30^ In this direction, we investigated the presence of biosynthetic gene cluster (BGCs) for the production of secondary metabolites, within the genome of wpSGBs. Such BGCs can produce a wide variety of natural products, including antibiotics, antifungals, and other bioactive compounds, with a possible relevant importance in host-protection.^31^ We found 34 BGCs encoded by 16 wpSGBs (Table 2). In particular, such secondary metabolites are ranthipeptides, arylpolyenes, terpenes, betalactones, thiopeptide, type I polyketides, non-ribosomal peptides (NRPs), ribosomally synthesized and post translationally modified peptides (RiPPs), and RiPP precursor peptide recognition elements (RREs). These molecules contain specific and broad-spectrum antimicrobials, plant-ground mediators, and molecules that participate in the microbial quorum sensing. In order to explore the global diffusion of such BGCs into the human gut metagenome, we screened the same metagenomes we previously used for the genes involved in wild plant food degradation. We found that the 34 BGCs are present into the gut metagenome of individuals relying on a rural lifestyle, with only few exceptions, mainly related to BGCs of the wpSGB assigned to *E. coli* (Figure 5B). Conversely, BGCs associated with the production of arylpolyenes, type I polyketides, terpenes, and lactones were exclusively present in the rural individuals, irrespective of their geographic origin, as if their presence were linked to the lifestyle. When looking at BGCs specifically present in our cohorts, we found that arylpolyenes produced by *Duodenibacillus sp900767875*, together with arylpolyenes and terpenes produced by *Merdousia sp002437405*, and BGCs for NRPs of the *Butyricicoccus A sp002395695* were very specific of Baka and Nzime individuals. We are here tempted to speculate that their presence could be connected to western African rainforest wild plant food ingestion and consumption.

**Table 2:** List of BGCs, wpSGBs, taxonomic assignments, and products

BGC ID	wpSGBs	Taxonomy	Product
BGC_1	BIZ06_bin.19	d__Bacteria; p__Firmicutes_A; c__Clostridia; o__Christensenellales; f__CAG-138; g__PeH17; s__PeH17 sp000435055	Ranthipeptide
BGC_2	BIZ06_bin.19	d__Bacteria; p__Firmicutes_A; c__Clostridia; o__Christensenellales; f__CAG-138; g__PeH17; s__PeH17 sp000435055	RRE-containing
BGC_3	BIZ06_bin.19	d__Bacteria; p__Firmicutes_A; c__Clostridia; o__Christensenellales; f__CAG-138; g__PeH17; s__PeH17 sp000435055	RRE-containing
BGC_4	BIZ06_bin.2	d__Bacteria; p__Firmicutes_A; c__Clostridia; o__Christensenellales; f__CAG-138; g__Phil1; s__Phil1 sp001940855	Ranthipeptide
BGC_5	BIZ06_bin.2	d__Bacteria; p__Firmicutes_A; c__Clostridia; o__Christensenellales; f__CAG-138; g__Phil1; s__Phil1 sp001940855	RRE-containing
BGC_6	BIZ06_bin.2	d__Bacteria; p__Firmicutes_A; c__Clostridia; o__Christensenellales; f__CAG-138; g__Phil1; s__Phil1 sp001940855	RRE-containing
BGC_7	BIZ07_bin.63	d__Bacteria; p__Proteobacteria; c__Gammaproteobacteria; o__Enterobacterales; f__Enterobacteriaceae; g__Escherichia; s__Escherichia coli	Thiopeptide
BGC_8	BIZ07_bin.63	d__Bacteria; p__Proteobacteria; c__Gammaproteobacteria; o__Enterobacterales; f__Enterobacteriaceae; g__Escherichia; s__Escherichia coli	NRPS
BGC_9	BIZ11_bin.5	d__Bacteria; p__Proteobacteria; c__Gammaproteobacteria; o__Burkholderiales; f__Burkholderiaceae; g__Duodenibacillus; s__Duodenibacillus sp900767875	Arylpolyene
BGC_10	BIZ12_bin.23	d__Bacteria; p__Proteobacteria; c__Gammaproteobacteria; o__Burkholderiales; f__Burkholderiaceae; g__Sutterella; s__	Arylpolyene
BGC_11	BIZ16_bin.15	d__Bacteria; p__Firmicutes_A; c__Clostridia; o__Oscillospirales; f__Ruminococcaceae; g__Ruminococcus_C; s__Ruminococcus_C sp900545285	Ranthipeptide
BGC_12	BIZ16_bin.15	d__Bacteria; p__Firmicutes_A; c__Clostridia; o__Oscillospirales; f__Ruminococcaceae; g__Ruminococcus_C; s__Ruminococcus_C sp900545285	Cyclic-lactone-autoinducer
BGC_13	BOS02_bin.33	d__Bacteria; p__Verrucomicrobiota; c__Verrucomicrobiae; o__Opitutales; f__CAG-312; g__Merdousia; s__Merdousia sp002437405	Terpene
BGC_14	BOS02_bin.33	d__Bacteria; p__Verrucomicrobiota; c__Verrucomicrobiae; o__Opitutales; f__CAG-312; g__Merdousia; s__Merdousia sp002437405	Arylpolyene
BGC_15	BOS03_bin.9	d__Bacteria; p__Elusimicrobiota; c__Elusimicrobia; o__Elusimicrobiales; f__Elusimicrobiaceae; g__UBA1436; s__UBA1436 sp900541355	T1PKS
BGC_16	BOS03_bin.9	d__Bacteria; p__Elusimicrobiota; c__Elusimicrobia; o__Elusimicrobiales; f__Elusimicrobiaceae; g__UBA1436; s__UBA1436 sp900541355	Terpene
BGC_17	BOS03_bin.9	d__Bacteria; p__Elusimicrobiota; c__Elusimicrobia; o__Elusimicrobiales; f__Elusimicrobiaceae; g__UBA1436; s__UBA1436 sp900541355	Terpene
BGC_18	BOS05_bin.27	d__Bacteria; p__Firmicutes_A; c__Clostridia; o__Oscillospirales; f__Ruminococcaceae; g__CAG-353; s__CAG-353 sp900768995	Ranthipeptide
BGC_19	BOS05_bin.8	d__Bacteria; p__Myxococcota; c__Bradymonadia; o__UBA4248; f__UBA4248; g__UBA4248; s__	RRE-containing
BGC_20	BOS11_bin.13	d__Bacteria; p__Proteobacteria; c__Gammaproteobacteria; o__Enterobacterales; f__Succinivibrionaceae; g__Succinivibrio; s__Succinivibrio sp000431835	Terpene
BGC_21	BOS11_bin.13	d__Bacteria; p__Proteobacteria; c__Gammaproteobacteria; o__Enterobacterales; f__Succinivibrionaceae; g__Succinivibrio; s__Succinivibrio sp000431835	Arylpolyene
BGC_22	BOS11_bin.13	d__Bacteria; p__Proteobacteria; c__Gammaproteobacteria; o__Enterobacterales; f__Succinivibrionaceae; g__Succinivibrio; s__Succinivibrio sp000431835	Betalactone
BGC_23	BOS11_bin.13	d__Bacteria; p__Proteobacteria; c__Gammaproteobacteria; o__Enterobacterales; f__Succinivibrionaceae; g__Succinivibrio; s__Succinivibrio sp000431835	Ranthipeptide
BGC_24	BOS12_bin.3	d__Bacteria; p__Spirochaetota; c__Spirochaetia; o__Treponematales; f__Treponemataceae; g__Treponema_D; s__Treponema_D sp900541945	RiPP-like
BGC_25	BOS12_bin.3	d__Bacteria; p__Spirochaetota; c__Spirochaetia; o__Treponematales; f__Treponemataceae; g__Treponema_D; s__Treponema_D sp900541945	Arylpolyene
BGC_26	BOS12_bin.3	d__Bacteria; p__Spirochaetota; c__Spirochaetia; o__Treponematales; f__Treponemataceae; g__Treponema_D; s__Treponema_D sp900541945	Arylpolyene
BGC_27	BOS12_bin.3	d__Bacteria; p__Spirochaetota; c__Spirochaetia; o__Treponematales; f__Treponemataceae; g__Treponema_D; s__Treponema_D sp900541945	RiPP-like
BGC_28	BOS14_bin.1	d__Bacteria; p__Firmicutes_A; c__Clostridia; o__Oscillospirales; f__Butyricicoccaceae; g__Butyricicoccus_A; s__Butyricicoccus_An sp002395695	Ranthipeptide
BGC_29	BOS14_bin.1	d__Bacteria; p__Firmicutes_A; c__Clostridia; o__Oscillospirales; f__Butyricicoccaceae; g__Butyricicoccus_A; s__Butyricicoccus_An sp002395695	RRE-containing
BGC_30	BOS14_bin.1	d__Bacteria; p__Firmicutes_A; c__Clostridia; o__Oscillospirales; f__Butyricicoccaceae; g__Butyricicoccus_A; s__Butyricicoccus_An sp002395695	NRPS
BGC_31	BOS14_bin.1	d__Bacteria; p__Firmicutes_A; c__Clostridia; o__Oscillospirales; f__Butyricicoccaceae; g__Butyricicoccus_A; s__Butyricicoccus_An sp002395695	NRPS-like
BGC_32	BOS21_bin.24	d__Bacteria; p__Firmicutes_A; c__Clostridia; o__Oscillospirales; f__Oscillospiraceae; g__CAG-83; s__CAG-83 sp000435975	RRE-containing
BGC_33	BOS24_bin.22	d__Bacteria; p__Firmicutes_A; c__Clostridia; o__Oscillospirales; f__Oscillospiraceae; g__Faecousia; s__Faecousia sp000434635	RRE-containing
BGC_34	BOS25_bin.44	d__Bacteria; p__Verrucomicrobiota; c__Kiritimatiellae; o__RFP12; f__UBA1067; g__RUG572; s__RUG572 sp900547945	Arylpolyene

## Discussion

Baka communities are increasingly faced with challenges to their culture and livelihood through influences such as land displacement for exploitation of the natural resources, government policies that favor agricultural societies, and climate change with drought that may disrupt traditional patterns of migration and make it difficult to find food and water resources.^20^ These favor the transition from foraging and small-scale cultivation to settled agriculture and industrialization, with an increase in consumption of processed foods and decrease in wild plant foods and game.^32^ Here, we demonstrated that the consumption of wild plant foods is associated with the presence of a specific microbial module of 26 wpSGBs, providing taxa and functionalities that are preserved almost in their entirety across other rural populations and lost in industrialized populations. Coherently, part of these microbes encoded for genes that are no longer detected in industrialized GM, such as urease, pullulanase, dihydrolipoyl dehydrogenase, sialidase, mannosyltransferase, and a protein assigned to the CBM57 module, together with BGCs for the production of arylpolyenes, polyketides, terpenes, NRPs, and lactones. Such enzymes are mainly involved in substrate degradation and signaling, whereas the secondary metabolites are more connected to the microbe-host crosstalk with unexplored effects on human host.

Specifically, the pullulanase catalyzes the hydrolysis of pullulan, a polysaccharide composed by maltotriose units, into smaller molecules, comprising glucose, and driving to the production of short-chain fatty acids (SCFAs) such as propionate, which have been shown to have anti-inflammatory properties and can help to promote gut health.^33^ Pullulanase-producing bacteria may thus play a role in the metabolism of dietary fiber and other complex carbohydrates, which can be difficult for humans to digest on their own. In particular, by breaking down these complex molecules, pullulanase-producing bacteria can help to release nutrients that would otherwise be inaccessible to the human body. When we considered the carbohydrate-binding module 57 (CBM57), we found that it is associated with bacterial enzymes involved in the breakdown of complex carbohydrates, including the plant-derived lignocellulose.^34^^,^^35^ Coherently, also mannosyltransferase was previously identified as one of the microbial CAZymes involved in the degradation of complex microbial polymers.^36^ Taken together, the presence of pullulanase, CBM57, and mannosyltransferase is consistent with the ingestion of wild plant foods, rich in fiber and complex carbohydrates, which are not absorbable by the human host and therefore potentially usable by bacteria possessing at least one of these three genes. The considerations about urease and sialidase are more complicated, because they are central enzymes in bacterial metabolism, usually involved in both degradation of substrates and microbe-host signaling.^37^^,^^38^^,^^39^ For this reason, further experiments are necessary to disentangle their peculiarities with respect to the analogous enzymes present in the industrial GM.

Interestingly, through the selection of the wpSGBs, western African rainforest wild plant foods would also result in the provision of a pool of wpSGBs-associated BGCs, being specific of our cohort and of other rural populations. Generally, we found that the associated secondary metabolites were important for the host protection, as antioxidant and antimicrobial against potential pathogens and as regulators of the microbiome-microbiome and microbiome-host interaction processes.^40^^,^^41^^,^^42^ We realized that these molecules, produced in the human gut, could have important effect on our health, as highlight in recent studies. For instance, a previous work conducted by Masyita et al. explored the potential role of terpenes and terpenoids in human health and food industry, showing their possible application as antianxiety, anticancer, anti-inflammatory, and analgesic molecules and also as antimicrobial and food preservative.^43^^,^^44^ The same can be sustained for betalactones, such as tetrahydrolipstatin and salinosporamide A, that have been described as molecules with potent bioactivity against bacteria, fungi, or human cancer cell lines.^45^ Also, type I polyketides, such as erythromycin and jamaicamide, are characterized by a diverse range of chemical structures and biological activities, and they have been the subject of extensive research for their potential use as antibiotics, anticancer agents, and other therapeutics.^46^ Finally, arylpolyenes increase protection from oxidative stress and contribute to biofilm formation, and for this reason its biosynthesis pathway has been explored to prevent biofilm formation of multidrug-resistant pathogens.^47^ It is tempting to speculate that the diversity of such bioactive secondary metabolites in the intestine of Baka and Nzime individuals—and other rural populations—may be an additional benefit coming from the consumption of wild plant foods, which, selecting for wpSGBs, will also provide for the associated and diverse pool of BGCs, possibly providing a range of bioactive metabolites in support for a better gut health. This hypothesis well combines with the low relevance of non-communicable diseases, including metabolic disorders and cancers, in such populations.^48^ However, such results need to be further investigated through the isolation of the specific bacteria and the characterization of the chemical structure of the secondary metabolites, for retrieving more insights on their contributions on human health.

Taken together, our results shed further light on the microbiome portion associated with the consumption of western African rainforest wild plant foods and traditional lifestyles, highlighting the genetic characteristics that this component carries in its genomes, with a particular attention to those genes that are no longer present in the microbiome of industrialized individuals. The work emphasizes the view of exploring microbiome diversity in traditional populations for identifying the important functionalities to be protected, as strategic for the extension of our phenotypic landscape,^49^ as providing the access to specific plant-based foods and also being important for keeping the gut homoeostasis, safeguarding our health. Further, by shedding light on unexplored services provided by the GM to humans who rely on a rural lifestyle consuming western African rainforest wild plant foods, we also have the opportunity to evaluate the impact of modernization on human GM and health. Finally, our work through the evidence of microbes containing BGCs whose presence is associated with the ingestion and/or gathering of wild plant foods nurtures the hypothesis that the GM biodiversity loss linked to industrialization may also be connected to eating predominantly processed and sterile industrial foods.

### Limitations of the study

Our study is limited by the small sample size of Baka and Nzime forager-horticulturalists, which limits the extensibility of our findings.

## STAR★Methods

### Key resources table

**Table:** 

REAGENT or RESOURCE	SOURCE	IDENTIFIER
**Critical commercial assays**		

DNeasy Blood & Tissue	QIAGEN	Cat#69506
NextSeq 500/550 High Output Kit v2.5 (300 Cycles)	Illumina	Cat#20024908
QIAseq FX DNA Library CDI Kit (96)	QIAGEN	Cat#180484
AMPure XP magnetic beads	Beckman Coulter	Cat#A63881

**Deposited data**		

Human gut metagenomes	Asnicar et al.^50^	Project number PRJNA339914
Human gut metagenomes	Bäckhed et al.^51^	Project number PRJEB6456
Human gut metagenomes	Conteville et al.^52^	Project number PRJNA527208
Human gut metagenomes	Costea et al.^53^	Project number PRJEB17632
Human gut metagenomes	Dhakan et al.^54^	Project number PRJNA397112
Human gut metagenomes	Obregon-Tito et al.^55^	Project number PRJNA268964
Human gut metagenomes	Qin et al.^56^	Project number PRJNA422434
Human gut metagenomes	Qin et al.^57^	Project number PRJEB6337
Human gut metagenomes	Rampelli et al.^6^	Project number PRJNA278393
Human gut metagenomes	Rampelli et al.^58^	Project number PRJNA553191
Human gut metagenomes	This study	Project number PRJEB63347
Human gut SGBs	This study	https://site.unibo.it/microbiome-science-biotechnology-unit/en/microbiome-materials-and-databases

**Software and algorithms**		

antiSMASH 6.0	Blin et al.^59^	https://github.com/antismash/antismash
bowtie2 2.3.4.3	Langmead et al.^60^	https://github.com/BenLangmead/bowtie2
CarveMe 1.5.1	Machado et al.^61^	https://github.com/cdanielmachado/carveme
CheckM 1.2.0	Parks et al.^62^	https://github.com/Ecogenomics/CheckM
CONCOCT 1.1.0	Alneberg et al.^63^	https://github.com/BinPro/CONCOCT
dbCAN-seq	Zheng et al.^64^	https://bcb.unl.edu/dbCAN_seq/
dRep 3.2.2	Olm et al.^65^	https://github.com/MrOlm/drep
gplots 3.1.3 r package	Warner et al.^66^	https://CRAN.R-project.org/package=gplots
GTDB-Tk 2.1.0	Chaumeil et al.^67^	https://github.com/Ecogenomics/GTDBTk
MaxBin 2.0	Wu et al.,^68^	https://sourceforge.net/projects/maxbin/
metabat2	Kang et al.^69^	https://bioconda.github.io/recipes/metabat2/README.html
Metage2metabo 1.5.0	Belcour et al.^26^	https://github.com/AuReMe/metage2metabo
metaSPAdes	Nurk et al.^70^	https://github.com/ablab/spades
Metawrap 1.3.2	Uritskiy et al.^71^	https://github.com/bxlab/metaWRAP
PhyloPhlAn 3.0.60	Asnicar et al.^72^	https://github.com/biobakery/phylophlan
Prokka 1.14.6	Seemann^73^	https://github.com/tseemann/prokka
reshape2 1.4.4 r package	Wickham, H.^74^	https://cran.r-project.org/web/packages/reshape2/index.html
Roary 3.13.0	Page et al.^75^	https://github.com/sanger-pathogens/Roary
RColorBrewer 1.1-3 package	Neuwirth, E.^76^	https://CRAN.R-project.org/package=RColorBrewer
R Software 4.2.0	R Software	www.r-project.org
Samtools 1.10	Bonfield et al.^77^	https://github.com/samtools/samtools
StrainPhlAn3	Truong et al.^28^	https://github.com/biobakery/MetaPhlAn/wiki/StrainPhlAn-3
tidyverse 1.3.2 r package	Wickhamn et al.^78^	https://cran.r-project.org/web/packages/tidyverse/index.html
vegan 2.6-2 r package	Oksanen et al.^79^	https://CRAN.R-project.org/package=vegan
viridis 0.6.2 r package	Garnier et al.^80^	https://cran.r-project.org/web/packages/viridis/index.html
XenoPath v 0.1	XenoPath	https://github.com/TessaTi/XenoPath

### Resource availability

#### Lead contact

Further information and request for resources and reagents should be directed to and will be fulfilled by the lead contacts, Marco Candela and Amanda Henry.

#### Materials availability

This study did not generate new unique reagents.

#### Data and code availability

• High-quality reads from the samples sequenced in this study were deposited and are publicly accessible in the European Nucleotide Archive under the project accession number PRJEB63347, SGBs are available here https://site.unibo.it/microbiome-science-biotechnology-unit/en/microbiome-materials-and-databases.
• This paper does not report original code.
• Any additional information required to reanalyze the data reported in this paper is available from the lead contact upon request.

### Experimental model and study participant details

#### Human metagenomes

Human metagenome datasets used in this study are sequenced in this study (see data and code availability section for further details) and derived from 9 previously published studies available in public repositories (see Table S5 for accession numbers). The latter included 927 subjects spanning different countries (USA, Peru, Sweden, Germany, Brazil, India, Italy, China and Tanzania) and lifestyles (industrial urban populations, hunter-gatherers and rural communities).

### Method details

#### Sample collection

The work is part of a broader study of foraging strategies among the Baka of southeastern Cameroon, with a specific focus on the importance of plant dietary foods (https://harvestproject.eu/). Fecal aliquots sequenced for this study come from 26 adult Baka volunteers, including 16 individuals from Kungu, a forest camp (Baka forest) and 10 individuals from Le Bosquet (Baka settlement), together with 18 individuals from the neighboring Nzime village, Nkeadinako (Nzime village). All individuals were healthy and had not received antibiotics for at least 3 months before sampling. Age, sex and when possible weight of the individuals are reported in Table S6. For sample collection and storage, we followed the same procedure reported in the work of Schnorr and colleagues (Schnorr et al.^3^). Briefly, samples were submerged in 97% ethanol for 24-36 h, after which ethanol was poured off and solid material was transferred to 50 ml tubes containing silica beads (Sigma 10087). Samples were stored at -80°C at all times upon arrival at the laboratory, until their processing. All work was approved by the ethics committee of Leipzig University (196–16/ek), and the Ethical Committee from the Ministry of Health of Cameroon (n°2018/06/1049/CE/CNERSH/SP) and received the research permit from the Ministry of Scientific Research and Innovation (00016/MINRESI/B00/C00/C10/C12). Before the onset of the study, we first obtained Free Prior and Informed Consents in all villages and from every individual participating in this study. Such consents included a detailed description of how the fecal samples would be used. Immediately after they provided samples, we analyzed subsamples of the feces to test for parasite eggs in the presence of the participant, showing the process under the microscope, and providing information about fecal parasites and their transmission. When we identified parasite eggs we informed the participant and instructed them to discuss it with the local medical service. In our final field season we returned to the communities and shared with them the preliminary results of the microbiome study. In this presentation we showed them the processing methods and photos of the team, and explained how their (community) GM was different from other communities that were studied previously.

#### DNA extraction and shotgun sequencing

Metagenomic DNA libraries were prepared using the QIAseq FX DNA Library Kit, following the manufacturer’s instructions (QIAGEN). Briefly, for each sample, 100 ng of DNA were fragmented to 450-bp size, end-repaired, and A-tailed using FX Enzyme Mix with the following thermal cycle: 4°C for 1 min, 32°C for 8 min, and 65°C for 30 min. Illumina adapter barcodes were attached through a 15-min incubation at 20°C in presence of the DNA ligase enzyme. After two purification steps with Agencourt AMPure XP magnetic beads (Beckman Coulter), 10-cycle PCR amplification, and a further step of purification as above, samples were pooled at equimolar concentration of 4 nM. Sequencing was performed on an Illumina NextSeq 500 platform using a 2 × 150 bp paired-end protocol, following the manufacturer’s instructions (Illumina). A sequencing control from DNA extraction to library preparation was performed and sequenced by 16S RNA sequencing on an Illumina platform to detect any contamination. Only 99 reads, mainly assigned to Aeromonadaceae and unclassified genus of the family *Lachnospiraceae* (47 and 32 reads, respectively), were detected by our analysis. (Table S7).

#### *Species-level genome bins (SGBs)* identification and analysis

Raw reads were filtered from human DNA and quality using the human sequence removal pipeline and the WGS read processing procedure of the Human Microbiome Project (HMP).^81^ High-quality reads were *de novo* assembled into longer sequences (contigs), and contigs were binned into metagenome assembled genomes (MAGs) using the metawrap pipeline,^71^ with metaspades,^70^ maxbin2,^68^ metabat2^69^ and concoct.^63^ Quality controls (completeness, contamination, genome size (bp), number of contigs, contig N50 values, mean contig length), were assessed using the lineage-specific workflow in CheckM with default settings and reported in Table S8.^62^ Only MAGs with a completeness above 50% and a contamination lower than 5% were retained and then dereplicated into Species-level genome bins (SGBs) using the dRep dereplicate command (dRep version 3.2.2)^65^ and the following parameters “--ignoreGenomeQuality -pa 0.90 -sa 0.95 -nc 0.30 -cm larger -centW 0”. GTDB-Tk was used for taxonomic assignment with default parameters.^67^ The abundance of SGBs in each sample was estimated by the metawrap quant_bins module^71^ and the genome annotation was retrieved by prokka^73^ using also the dbCAN^82^ and XenoPath databases (https://github.com/TessaTi/XenoPath). The sharing of genes across SGBs were determined using roary,^75^ with the following parameters “-I 90 -cd 17 -e -g 1000000”. A phylogenetic tree including all the SGBs were built by applying phylophlan^72^ with the default parameters and used for measuring UniFrac distances among samples in PCoA analysis.

#### Detection of strain-sharing events between individuals of the Baka settled and Nzime village groups working in the same fields

To gain a deeper insight into potential sharing of microbiome components across human metagenomes, we looked at the strain level population structure using StrainPhlAn3 as previously illustrated.^83^ We perform the analysis on the most abundant wpSGBs, i.e. those that are represented by at least 5 MAGs and whose abundance was > 5 gcpm (genome copies per million reads) in at least one individual from both Baka settled and Nzime village groups. For each species analyzed, custom wpSGB marker databases were constructed, by firstly selecting the core genes for each specific wpSGB from the roary output (i.e., the genes that were present only in the examined SGB and absent in the rest of the dataset). The MAGs comprised within each specific wpSGB were divided into 150 nucleotide fragments and aligned against their core genes using bowtie2 (version 2.3.4.3; --sensitive option). A core gene was considered valuable as marker genes for a wpSGB if at least 90% of MAGs mapped against it by covering >50% of the gene's length. To infer strain sharing, strain-level phylogenies were then reconstructed using bowtie2 (--sensitive option) and StrainPhlAn3 with parameters "--marker_in_n_samples 10 - -phylophlan_mode accurate" and the parameter “--sample_with_n_markers” set for retaining only samples with at least 10 marker genes.

To detect strain-sharing events, we first set wpSGB-specific normalized phylogenetic distance (nGD) thresholds that optimally separated same-group strain retention (same strain) from unrelated-individuals (different strain) nGD distributions (to this purpose we compared Baka – settled metagenomes with data from a previous study characterizing the microbiomes of the Hadza from Tanzania^6^). nGDs were calculated as leaf-to-leaf branch lengths normalized by total tree branch length in phylogenetic trees produced by StrainPhlAn, which are built on marker gene alignments. nGD thresholds were then defined based on maximizing Youden’s index and limiting at 5% the fraction of unrelated individuals to share the same strain as a bound on a false discovery rate.

#### Genome scale metabolic models for western African rainforest wild plant foods substrate degradation

Microbiome-scale metabolic complementarity for the identification of key species devoted to the degradation of wild plant food substrates were obtained by applying carveme^61^ and Metage2Metabo.^26^ Specifically, carveme has been applied to each SGBs using the prokka outputs (.faa files) as input and the default options, in order to build the specific genome scale metabolic model (GSMM) for each SGBs. Metage2Metabo, with the command “metacom”, were used for creating a single metabolic network combining all the GSMM and retrieving the list of SGBs essential for the degradation of the western African rainforest wild plant foods (wpSGBs). In particular, for each western African rainforest wild plant foods, including *Gnetum africanum*, *Irvingia* spp., *Baillonella toxisperma*, *Panda oleosa* and *Afrostyrax lepidophyllus*, the command was repeated by providing as input the complete set of GSMMs and the metabolic composition of the western African rainforest wild plant food from recent publications.^84^^,^^85^^,^^86^^,^^87^^,^^88^ The list of the wpSGBs was compiled by selecting the bacteria that are involved in the metabolism of at least one of these wild plant foods.

#### Spread of wpSGB features in the global populations

Further examining the shared features from the output of roary, we identified those genes that were not contained in other SGBs, but only specifically present in wpSGBs. From these genes, we selected those annotated in the dbCAN or XenoPath databases, because of interest for the degradation of plant substrates. We then applied antismash 6.0^59^ to the wpSGBs, for selecting eventual BGCs that were potentially connected to plant consumption or involved in microbe-plant crosstalk. The identified features were used to build a database, to which 970 metagenomes from populations from all over the world (Table S5) were aligned using bowtie2 with the --end-to-end tag.^60^ The number of aligned reads for each sample was retrieved using samtools^77^ and normalized for sequencing depth and length of the references, obtaining reads per kilobase of genes per million reads mapped (RPKM) as unit of measurement.

### Quantification and statistical analysis

#### Biostatistics and graphical representation

All statistical analysis and graphical representation were performed using the R software (v. 4.2.0, www.r-project.org) with packages vegan (version 2.6-2),^79^ RColorBrewer (version 1.1-3),^76^ gplots (version 3.1.3),^66^ viridis (version 0.6.2),^80^ reshape2 (version 1.4.4),^74^ tidyverse (version 1.3.2).^78^ Data separation in the Principal Coordinates Analysis (PCoA) was evaluated using a permutation test with pseudo-F ratios (function adonis in the vegan package). Kruskall-Wallis test was used to assess significant differences between groups. p values, when necessary, were corrected for multiple testing by means of the Benjamini-Hochberg method, with a false discovery rate (FDR) ≤ 0.05 considered to be statistically significant.

## Supplemental information

Document S1. Figures S1–S3 and Tables S1, S3, S6, and S7

Table S2. List of previously known SGBs, relative references and MASH distance, related to Figures 3 and 4 This file is submitted separately, due to its dimension.

Table S4. SGB abundances, taxonomic assignments and clustering within our cohort, related to Figure 4

Table S5. List of 970 sample IDs and relative metadata, including the accession numbers in SRA and ENA websites, utilized in this study, related to Figure 5

Table S8. List of 161 SGBs with genome length, N50, completeness and contamination as predicted by checkM, related to Figures 3 and 4

## References

[1] De Filippo C., Cavalieri D., Di Paola M., Ramazzotti M., Poullet J.B., Massart S., Collini S., Pieraccini G., Lionetti P. (2010). Impact of diet in shaping gut microbiota revealed by a comparative study in children from Europe and rural Africa. Proc. Natl. Acad. Sci. USA.

[2] Yatsunenko T., Rey F.E., Manary M.J., Trehan I., Dominguez-Bello M.G., Contreras M., Magris M., Hidalgo G., Baldassano R.N., Anokhin A.P. (2012). Human gut microbiome viewed across age and geography. Nature.

[3] Schnorr S.L., Candela M., Rampelli S., Centanni M., Consolandi C., Basaglia G., Turroni S., Biagi E., Peano C., Severgnini M. (2014). Gut microbiome of the Hadza hunter-gatherers. Nat. Commun..

[4] Obregon-Tito A.J., Tito R.Y., Metcalf J., Sankaranarayanan K., Clemente J.C., Ursell L.K., Zech Xu Z., Van Treuren W., Knight R., Gaffney P.M. (2015). Subsistence strategies in traditional societies distinguish gut microbiomes. Nat. Commun..

[5] Martínez I., Stegen J.C., Maldonado-Gómez M.X., Eren A.M., Siba P.M., Greenhill A.R., Walter J. (2015). The Gut Microbiota of Rural Papua New guineans: composition, diversity patterns, and ecological processes. Cell Rep..

[6] Rampelli S., Schnorr S.L., Consolandi C., Turroni S., Severgnini M., Peano C., Brigidi P., Crittenden A.N., Henry A.G., Candela M. (2015). Metagenome Sequencing of the Hadza Hunter-Gatherer Gut Microbiota. Curr. Biol..

[7] Gomez A., Petrzelkova K.J., Burns M.B., Yeoman C.J., Amato K.R., Vlckova K., Modry D., Todd A., Jost Robinson C.A., Remis M.J. (2016). Gut Microbiome of Coexisting BaAka Pygmies and Bantu Reflects Gradients of Traditional Subsistence Patterns. Cell Rep..

[8] Jha A.R., Davenport E.R., Gautam Y., Bhandari D., Tandukar S., Ng K.M., Fragiadakis G.K., Holmes S., Gautam G.P., Leach J. (2018). Gut microbiome transition across a lifestyle gradient in Himalaya. PLoS Biol..

[9] Ayeni F.A., Biagi E., Rampelli S., Fiori J., Soverini M., Audu H.J., Cristino S., Caporali L., Schnorr S.L., Carelli V. (2018). Infant and Adult Gut Microbiome and Metabolome in Rural Bassa and Urban Settlers from Nigeria. Cell Rep..

[10] Smits S.A., Leach J., Sonnenburg E.D., Gonzalez C.G., Lichtman J.S., Reid G., Knight R., Manjurano A., Changalucha J., Elias J.E. (2017). Seasonal cycling in the gut microbiome of the Hadza hunter-gatherers of Tanzania. Science.

[11] Hansen M.E.B., Rubel M.A., Bailey A.G., Ranciaro A., Thompson S.R., Campbell M.C., Beggs W., Dave J.R., Mokone G.G., Mpoloka S.W. (2019). Population structure of human gut bacteria in a diverse cohort from rural Tanzania and Botswana. Genome Biol..

[12] Tamburini F.B., Maghini D., Oduaran O.H., Brewster R., Hulley M.R., Sahibdeen V., Norris S.A., Tollman S., Kahn K., Wagner R.G. (2022). Short- and long-read metagenomics of urban and rural South African gut microbiomes reveal a transitional composition and undescribed taxa. Nat. Commun..

[13] de Goffau M.C., Jallow A.T., Sanyang C., Prentice A.M., Meagher N., Price D.J., Revill P.A., Parkhill J., Pereira D.I.A., Wagner J. (2022). Gut microbiomes from Gambian infants reveal the development of a non-industrialized Prevotella-based trophic network. Nat. Microbiol..

[14] Manara S., Selma-Royo M., Huang K.D., Asnicar F., Armanini F., Blanco-Miguez A., Cumbo F., Golzato D., Manghi P., Pinto F. (2023). Maternal and food microbial sources shape the infant microbiome of a rural Ethiopian population. Curr. Biol..

[15] Rook G.A.W., Raison C.L., Lowry C.A. (2014). Microbial 'old friends', immunoregulation and socioeconomic status. Clin. Exp. Immunol..

[16] Sonnenburg J.L., Sonnenburg E.D. (2019). Vulnerability of the industrialized microbiota. Science.

[17] Candela M., Biagi E., Maccaferri S., Turroni S., Brigidi P. (2012). Intestinal microbiota is a plastic factor responding to environmental changes. Trends Microbiol..

[18] Sonnenburg E.D., Sonnenburg J.L. (2019). The ancestral and industrialized gut microbiota and implications for human health. Nat. Rev. Microbiol..

[19] Bello M.G.D., Knight R., Gilbert J.A., Blaser M.J. (2018). Preserving microbial diversity. Science.

[20] Gallois S., Henry A.G. (2021). The Cost of Gathering Among the Baka Forager-Horticulturalists From Southeastern Cameroon. Front. Ecol. Evol..

[21] Reyes-García V., Powell B., Díaz-Reviriego I., Fernández-Llamazares Á., Gallois S., Gueze M. (2019). Dietary transitions among three contemporary hunter-gatherers across the tropics. Food Secur..

[22] Gallois S., Heger T., van Andel T., Sonké B., Henry A.G. (2020). From Bush Mangoes to Bouillon Cubes: Wild Plants and Diet among the Baka, Forager-Horticulturalists from Southeast Cameroon. Econ. Bot..

[23] Gallois S., van Andel T., Heger T., Sonké B., Henry A.G. (2020). Comparing Apples and Pears: the Hidden Diversity of Central African Bush Mangoes (Irvingiaceae). Econ. Bot..

[24] Gallois S., Heger T., Henry A.G., van Andel T. (2021). The importance of choosing appropriate methods for assessing wild food plant knowledge and use: A case study among the Baka in Cameroon. PLoS One.

[25] Pasolli E., Asnicar F., Manara S., Zolfo M., Karcher N., Armanini F., Beghini F., Manghi P., Tett A., Ghensi P. (2019). Extensive Unexplored Human Microbiome Diversity Revealed by Over 150,000 Genomes from Metagenomes Spanning Age, Geography, and Lifestyle. Cell.

[26] Belcour A., Frioux C., Aite M., Bretaudeau A., Hildebrand F., Siegel A. (2020). Metage2Metabo, microbiota-scale metabolic complementarity for the identification of key species. Elife.

[27] Rampelli S., Turroni S., Mallol C., Hernandez C., Galván B., Sistiaga A., Biagi E., Astolfi A., Brigidi P., Benazzi S. (2021). Components of a Neanderthal gut microbiome recovered from fecal sediments from El Salt. Commun. Biol..

[28] Truong D.T., Tett A., Pasolli E., Huttenhower C., Segata N. (2017). Microbial strain-level population structure and genetic diversity from metagenomes. Genome Res..

[29] Lucke M., Correa M.G., Levy A. (2020). The Role of Secretion Systems, Effectors, and Secondary Metabolites of Beneficial Rhizobacteria in Interactions With Plants and Microbes. Front. Plant Sci..

[30] Narayanan Z., Glick B.R. (2022). Secondary Metabolites Produced by Plant Growth-Promoting Bacterial Endophytes. Microorganisms.

[31] Ruiz B., Chávez A., Forero A., García-Huante Y., Romero A., Sánchez M., Rocha D., Sánchez B., Rodríguez-Sanoja R., Sánchez S., Langley E. (2010). Production of microbial secondary metabolites: regulation by the carbon source. Crit. Rev. Microbiol..

[32] Kuhnlein H.V. (2015).

[33] Flint H.J., Scott K.P., Duncan S.H., Louis P., Forano E. (2012). Microbial degradation of complex carbohydrates in the gut. Gut Microb..

[34] Do T.H., Le N.G., Dao T.K., Nguyen T.M.P., Le T.L., Luu H.L., Nguyen K.H.V., Nguyen V.L., Le L.A., Phung T.N. (2018). Metagenomic insights into lignocellulose-degrading genes through Illumina-based *de novo* sequencing of the microbiome in Vietnamese native goats' rumen. J. Gen. Appl. Microbiol..

[35] Lam M.Q., Oates N.C., Thevarajoo S., Tokiman L., Goh K.M., McQueen-Mason S.J., Bruce N.C., Chong C.S. (2020). Genomic analysis of a lignocellulose degrading strain from the underexplored genus Meridianimaribacter. Genomics.

[36] Costa O.Y.A., de Hollander M., Pijl A., Liu B., Kuramae E.E. (2020). Cultivation-independent and cultivation-dependent metagenomes reveal genetic and enzymatic potential of microbial community involved in the degradation of a complex microbial polymer. Microbiome.

[37] Mora D., Arioli S. (2014). Microbial urease in health and disease. PLoS Pathog..

[38] Juge N., Tailford L., Owen C.D. (2016). Sialidases from gut bacteria: a mini-review. Biochem. Soc. Trans..

[39] Cohen L.J., Han S.M., Lau P., Guisado D., Liang Y., Nakashige T.G., Ali T., Chiang D., Rahman A., Brady S.F. (2022). Unraveling function and diversity of bacterial lectins in the human microbiome. Nat. Commun..

[40] Schöner T.A., Gassel S., Osawa A., Tobias N.J., Okuno Y., Sakakibara Y., Shindo K., Sandmann G., Bode H.B. (2016). Aryl Polyenes, a Highly Abundant Class of Bacterial Natural Products, Are Functionally Related to Antioxidative Carotenoids. Chembiochem.

[41] Huang A.C., Jiang T., Liu Y.X., Bai Y.C., Reed J., Qu B., Goossens A., Nützmann H.W., Bai Y., Osbourn A. (2019). A specialized metabolic network selectively modulates Arabidopsis root microbiota. Science.

[42] Polturak G., Osbourn A. (2021). The emerging role of biosynthetic gene clusters in plant defense and plant interactions. PLoS Pathog..

[43] Araruna M.E., Serafim C., Alves Júnior E., Hiruma-Lima C., Diniz M., Batista L. (2020). Intestinal Anti-Inflammatory Activity of Terpenes in Experimental Models (2010-2020): A Review. Molecules.

[44] Masyita A., Mustika Sari R., Dwi Astuti A., Yasir B., Rahma Rumata N., Emran T.B., Nainu F., Simal-Gandara J. (2022). Terpenes and terpenoids as main bioactive compounds of essential oils, their roles in human health and potential application as natural food preservatives. Food Chem. X.

[45] Robinson S.L., Christenson J.K., Wackett L.P. (2019). Biosynthesis and chemical diversity of _-lactone natural products. Nat. Prod. Rep..

[46] Keatinge-Clay A.T. (2012). The structures of type I polyketide synthases. Nat. Prod. Rep..

[47] Johnston I., Osborn L.J., Markley R.L., McManus E.A., Kadam A., Schultz K.B., Nagajothi N., Ahern P.P., Brown J.M., Claesen J. (2021). Identification of essential genes for Escherichia coli aryl polyene biosynthesis and function in biofilm formation. NPJ Biofilms Microbiomes.

[48] GBD 2015 Mortality and Causes of Death Collaborators (2016). Global, regional, and national life expectancy, all-cause mortality, and cause-specific mortality for 249 causes of death, 1980-2015: A systematic analysis for the Global Burden of Disease Study 2015. Lancet.

[49] Henry L.P., Bruijning M., Forsberg S.K.G., Ayroles J.F. (2021). The microbiome extends host evolutionary potential. Nat. Commun..

[50] Asnicar F., Manara S., Zolfo M., Truong D.T., Scholz M., Armanini F., Ferretti P., Gorfer V., Pedrotti A., Tett A., Segata N. (2017). Studying Vertical Microbiome Transmission from Mothers to Infants by Strain-Level Metagenomic Profiling. mSystems.

[51] Bäckhed F., Roswall J., Peng Y., Feng Q., Jia H., Kovatcheva-Datchary P., Li Y., Xia Y., Xie H., Zhong H. (2015). Dynamics and Stabilization of the Human Gut Microbiome during the First Year of Life. Cell Host Microbe.

[52] Conteville L.C., Oliveira-Ferreira J., Vicente A.C.P. (2019). Gut Microbiome Biomarkers and Functional Diversity Within an Amazonian Semi-Nomadic Hunter-Gatherer Group. Front. Microbiol..

[53] Costea P.I., Coelho L.P., Sunagawa S., Munch R., Huerta-Cepas J., Forslund K., Hildebrand F., Kushugulova A., Zeller G., Bork P. (2017). Subspecies in the global human gut microbiome. Mol. Syst. Biol..

[54] Dhakan D.B., Maji A., Sharma A.K., Saxena R., Pulikkan J., Grace T., Gomez A., Scaria J., Amato K.R., Sharma V.K. (2019). The unique composition of Indian gut microbiome, gene catalogue, and associated fecal metabolome deciphered using multi-omics approaches. GigaScience.

[55] Obregon-Tito A.J., Tito R.Y., Metcalf J., Sankaranarayanan K., Clemente J.C., Ursell L.K., Zech, Xu Z., Van Treuren W., Knight R., Gaffney P.M. (2015). Subsistence strategies in traditional societies distinguish gut microbiomes. Nat. Commun..

[56] Qin J., Li Y., Cai Z., Li S., Zhu J., Zhang F., Liang S., Zhang W., Guan Y., Shen D. (2012). A metagenome-wide association study of gut microbiota in type 2 diabetes. Nature.

[57] Qin N., Yang F., Li A., Prifti E., Chen Y., Shao L., Guo J., Le Chatelier E., Yao J., Wu L. (2014). Alterations of the human gut microbiome in liver cirrhosis. Nature.

[58] Rampelli S., Soverini M., D’Amico F., Barone M., Tavella T., Monti D., Capri M., Astolfi A., Brigidi P., Biagi E. (2020). Shotgun Metagenomics of Gut Microbiota in Humans with up to Extreme Longevity and the Increasing Role of Xenobiotic Degradation. mSystems.

[59] Blin K., Shaw S., Kloosterman A.M., Charlop-Powers Z., van Wezel G.P., Medema M.H., Weber T. (2021). antiSMASH 6.0: improving cluster detection and comparison capabilities. Nucleic Acids Res..

[60] Langmead B., Salzberg S.L. (2012). Fast gapped-read alignment with Bowtie 2. Nat. Methods.

[61] Machado D., Andrejev S., Tramontano M., Patil K.R. (2018). Fast automated reconstruction of genome-scale metabolic models for microbial species and communities. Nucleic Acids Res..

[62] Parks D.H., Imelfort M., Skennerton C.T., Hugenholtz P., Tyson G.W. (2015). CheckM: assessing the quality of microbial genomes recovered from isolates, single cells, and metagenomes. Genome Res..

[63] Alneberg J., Bjarnason B.S., de Bruijn I., Schirmer M., Quick J., Ijaz U.Z., Lahti L., Loman N.J., Andersson A.F., Quince C. (2014). Binning metagenomic contigs by coverage and composition. Nat. Methods.

[64] Zheng J., Ge Q., Yan Y., Zhang X., Huang L., Yin Y. (2023). dbCAN3: automated carbohydrate-active enzyme and substrate annotation. Nucleic Acids Res..

[65] Olm M.R., Brown C.T., Brooks B., Banfield J.F. (2017). dRep: a tool for fast and accurate genomic comparisons that enables improved genome recovery from metagenomes through de-replication. ISME J..

[66] Warnes G., Bolker B., Bonebakker L., Gentleman R., Huber W., Liaw A., Lumley T., Maechler M., Magnusson A., Moeller S. (2022). https://CRAN.R-project.org/package=gplots.

[67] Chaumeil P.A., Mussig A.J., Hugenholtz P., Parks D.H. (2022). GTDB-Tk v2: memory friendly classification with the genome taxonomy database. Bioinformatics.

[68] Wu Y.W., Simmons B.A., Singer S.W. (2016). MaxBin 2.0: an automated binning algorithm to recover genomes from multiple metagenomic datasets. Bioinformation.

[69] Kang D.D., Li F., Kirton E., Thomas A., Egan R., An H., Wang Z. (2019). MetaBAT 2: an adaptive binning algorithm for robust and efficient genome reconstruction from metagenome assemblies. PeerJ.

[70] Nurk S., Meleshko D., Korobeynikov A., Pevzner P.A. (2017). metaSPAdes: a new versatile metagenomic assembler. Genome Res..

[71] Uritskiy G.V., DiRuggiero J., Taylor J. (2018). MetaWRAP-a flexible pipeline for genome-resolved metagenomic data analysis. Microbiome.

[72] Asnicar F., Thomas A.M., Beghini F., Mengoni C., Manara S., Manghi P., Zhu Q., Bolzan M., Cumbo F., May U. (2020). Precise phylogenetic analysis of microbial isolates and genomes from metagenomes using PhyloPhlAn 3.0. Nat. Commun..

[73] Seemann T. (2014). Prokka: rapid prokaryotic genome annotation. Bioinformatics.

[74] Wickham H. (2007). Reshaping data with the reshape package. J. Stat. Software.

[75] Page A.J., Cummins C.A., Hunt M., Wong V.K., Reuter S., Holden M.T.G., Fookes M., Falush D., Keane J.A., Parkhill J. (2015). Roary: rapid large-scale prokaryote pan genome analysis. Bioinformation.

[76] Neuwirth E. (2022). https://CRAN.R-project.org/package=RColorBrewer.

[77] Bonfield J.K., Marshall J., Danecek P., Li H., Ohan V., Whitwham A., Keane T., Davies R.M. (2021). HTSlib: C library for reading/writing high-throughput sequencing data. GigaScience.

[78] Wickham H., Averick M., Bryan J., Chang W., McGowan L., François R., Grolemund G., Hayes A., Henry L., Hester J. (2019). Welcome to the tidyverse. J. Open Source Softw..

[79] Oksanen J., Simpson G., Blanchet F., Kindt R., Legendre P., Minchin P., O'Hara R., Solymos P., Stevens M., Szoecs E. (2022). https://CRAN.R-project.org/package=vegan.

[80] Garnier S., Ross N., Rudis R., Camargo A.P., Sciaini M., Scherer C. (2021).

[81] Turnbaugh P.J., Ley R.E., Hamady M., Fraser-Liggett C.M., Knight R., Gordon J.I. (2007). The human microbiome project. Nature.

[82] Zheng J., Hu B., Zhang X., Ge Q., Yan Y., Akresi J., Piyush V., Huang L., Yin Y. (2023). dbCAN-seq update: CAZyme gene clusters and substrates in microbiomes. Nucleic Acids Res..

[83] Valles-Colomer M., Blanco-Míguez A., Manghi P., Asnicar F., Dubois L., Golzato D., Armanini F., Cumbo F., Huang K.D., Manara S. (2023). The person-to-person transmission landscape of the gut and oral microbiomes. Nature.

[84] Ali F., Assanta M.A., Robert C. (2011). Gnetum africanum: a wild food plant from the African forest with many nutritional and medicinal properties. J. Med. Food.

[85] Ogunsina B.S., Bhatnagar A.S., Indira T.N., Radha C. (2012). The proximate composition of African Bush Mango kernels (Irvingia gabonensis) and characteristics of its oil. IFE J. Sci..

[86] Fungo R., Muyonga J., Kaaya A., Okia C., Tieguhong J.C., Baidu-Forson J.J. (2015). Nutrients and bioactive compounds content of Baillonella toxisperma, Trichoscypha abut and Pentaclethra macrophylla from Cameroon. Food Sci. Nutr..

[87] Ngenge T.A., Jabeen A., Maurice T.F., Baig T.A., Shaheen F. (2019). Organic and Mineral Composition of Seeds of Afrostyrax lepidophyllus Mildbr. and Evaluation of ROS Inhibition and Cytotoxicity of Isolated Compounds. Chem. Afr..

[88] Fungo R., Muyonga J., Ngondi J., Mikolo-Yobo C., Iponga D., Ngoye A., Nchuaji Tang E., Chupezi Tieguhong J. (2019). Nutrient and Bioactive Composition of Five Gabonese Forest Fruits and Their Potential Contribution to Dietary Reference Intakes of Children Aged 1–3 Years and Women Aged 19–60 Years. Forests.

