# Supplementary material for: The gut microbiome of Baka forager-horticulturalists from Cameroon is optimized for wild plant foods
Source: iScience. 2024 Feb 10;27(3):109211. doi: 10.1016/j.isci.2024.109211 (PMC10904984; doi:10.1016/j.isci.2024.109211)
Supplement: Document S1. Figures S1–S3 and Tables S1, S3, S6, and S7 [file mmc1.pdf]

iScience, Volume *27*

Supplemental information

The gut microbiome of Baka forager-horticulturalists from Cameroon is optimized for wild plant foods

Simone Rampelli, Sandrine Gallois, Federica D'Amico, Silvia Turroni, Marco Fabbrini, Daniel Scicchitano, Marco Candela, and Amanda Henry

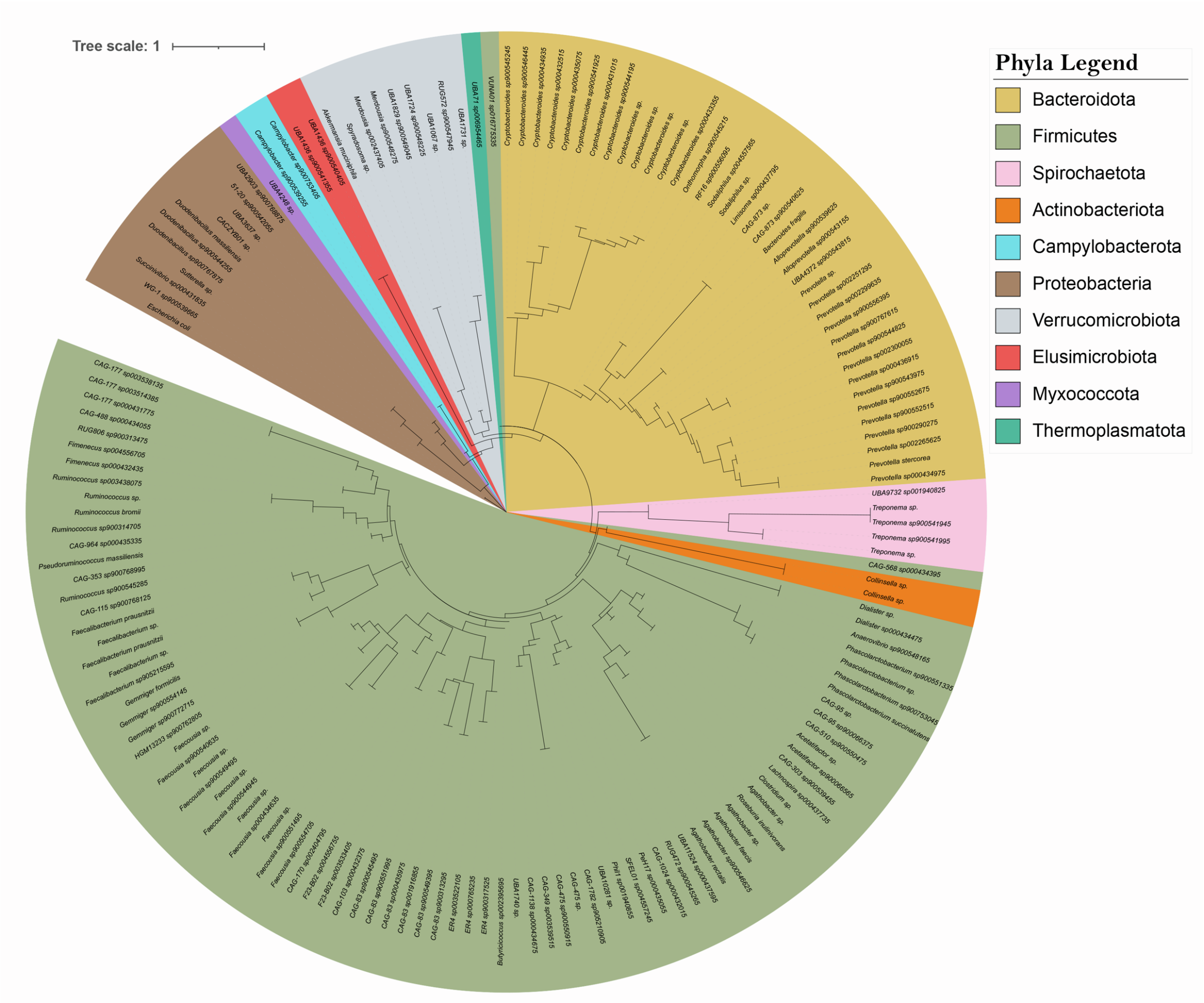

Figure S1. Related to Figure 2. Phylogenetic tree of 161 SGBs characterized in the framework of this study. The tree was built using PhyloPhlAn3.

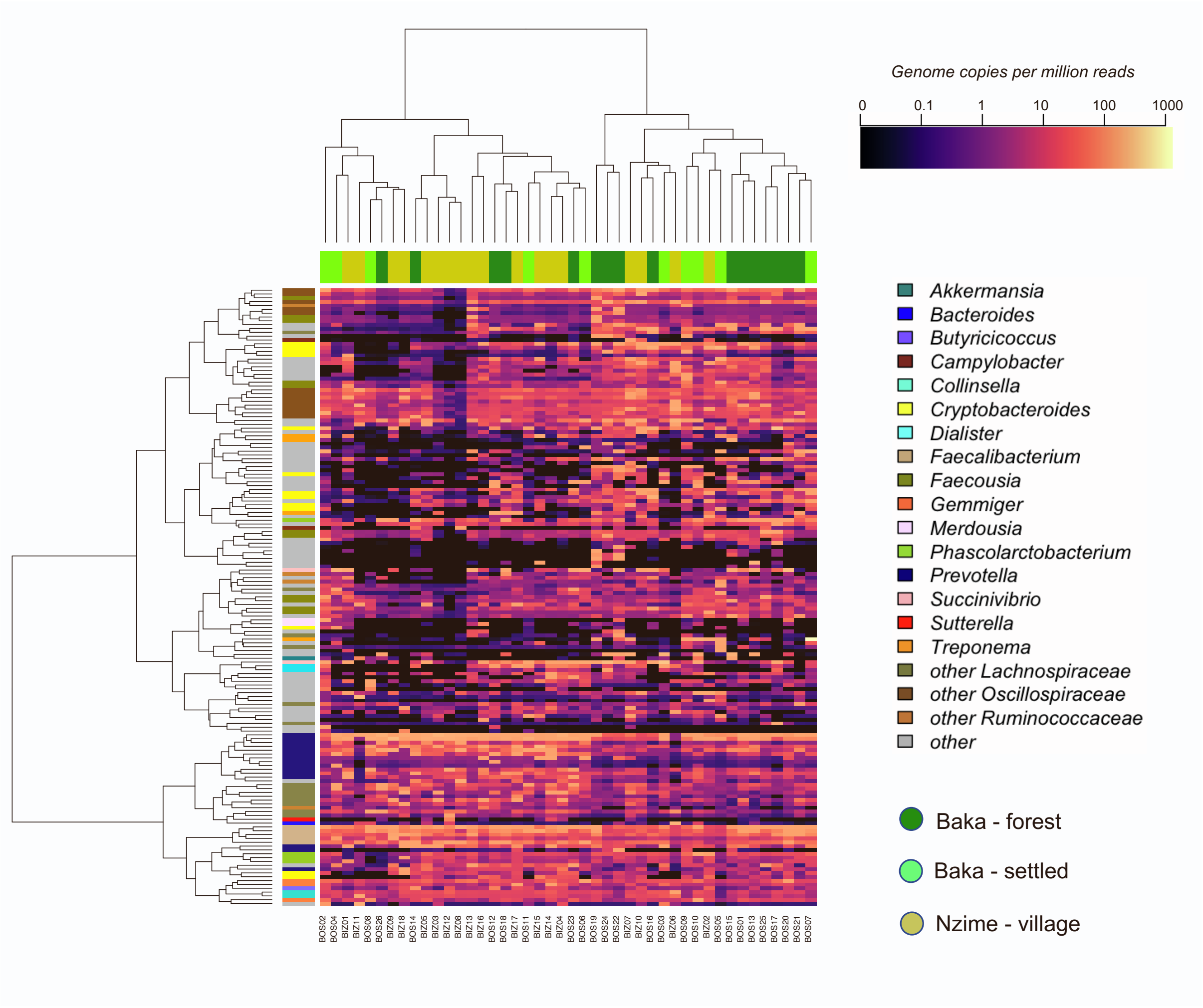

Figure S2. Related to Figure 2. Hierarchical Ward-linkage clustering based on the Spearman correlation coefficients of the SGB genome copies per million reads. 161 SGBs confidently classified at taxonomic level are clustered by the vertical tree.

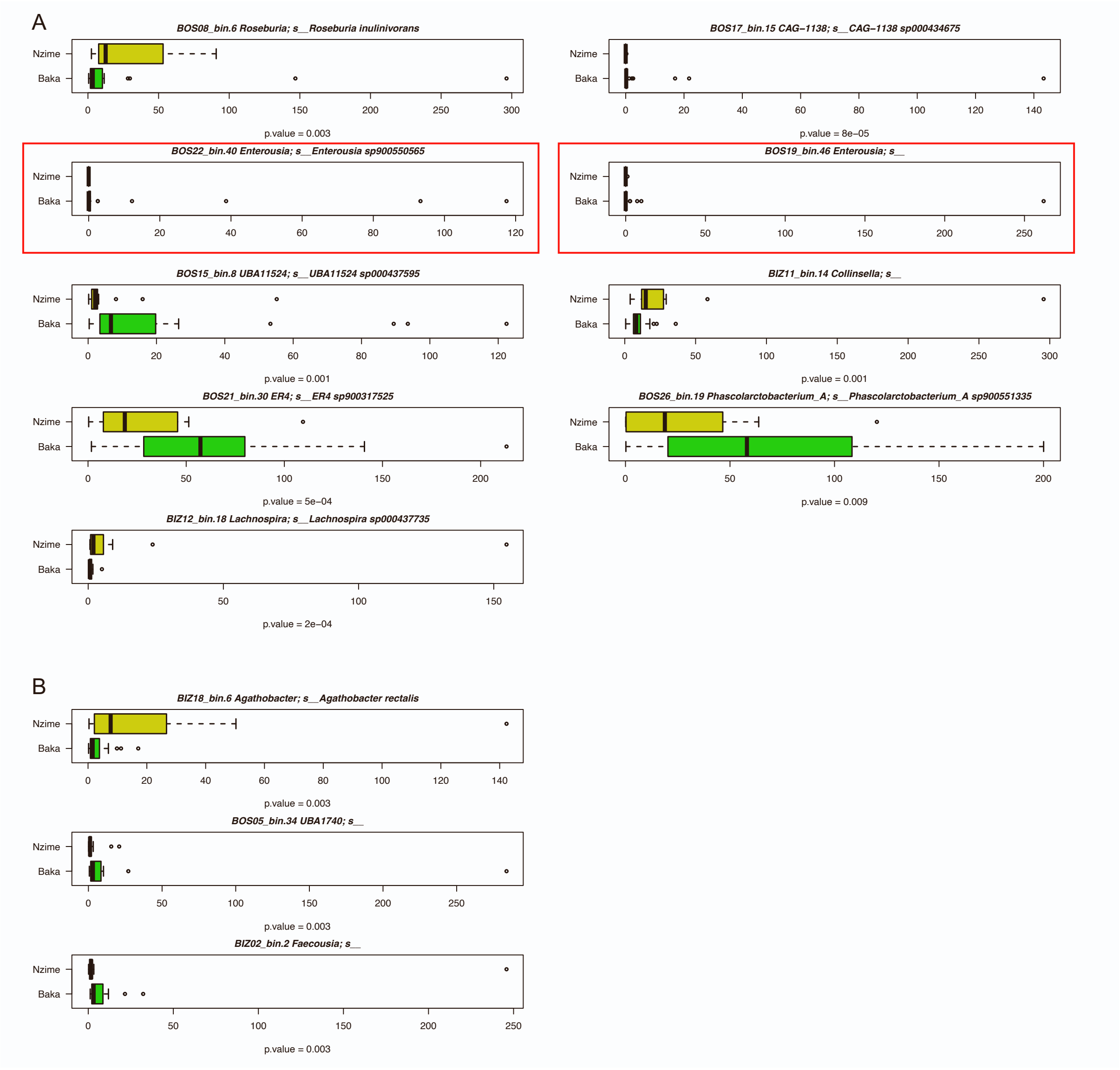

Figure S3. Related to Figure 2. Differences in SGB composition between individuals of the Baka (green) and Nzime (dark yellow) groups. (A) Box plots for SGB abundances, expressed as genome copies per million sequenced reads. The same SGBs as in Figure 2 are shown. Red squares are used to highlight those taxa whose abundances are not differentially distributed between the two groups after combining Baka Forest and Baka Settlement. (B) Three additional SGBs were found to be significantly differentially distributed between Baka and Nzime. P values are obtained using a Kruskall Wallis test.

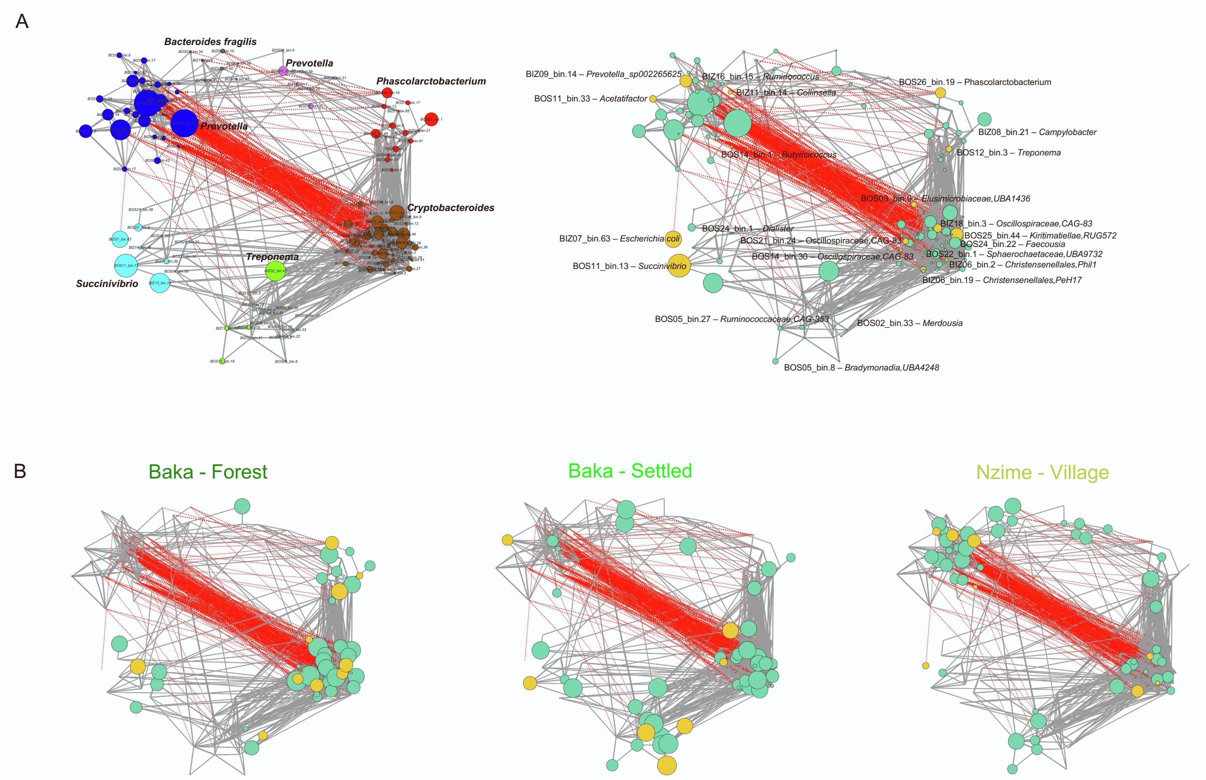

Figure S4. Related to Figure 4. Distinct bacterial networks characterized the three groups. (A) Bacterial network layout as obtained by Cytoscape, comprising all the SGBs. Only significant Kendall's tau correlation coefficients were considered. The leading taxa in each network are highlighted. A positive correlation is shown with a gray line and a negative correlation with a red line. Disc size is proportional to the mean relative abundance in the whole cohort. Discs are colored as in A (left), or on the basis of whether or not the SGBs was part of the essential taxa necessary for western African rainforest wild plant food degradation (right; wpSGBs in gold; other SGBs in green). (B) Network plots corresponding to the three groups from the whole cohort analysis, in which disc sizes indicate SGB over-abundance compared to the average abundance in the whole cohort. wpSGBs in gold; other SGBs in green.

Table S1. Related to Figure 2-5. Sequencing depth per sample.

ID	N seq	Group
**BIZ01**	9,487,437	Nzime Village
**BIZ02**	8,794,532	Nzime Village
**BIZ03**	8,696,876	Nzime Village
**BIZ04**	9,403,895	Nzime Village
**BIZ05**	9,630,668	Nzime Village
**BIZ06**	6,865,867	Nzime Village
**BIZ07**	7,210,782	Nzime Village
**BIZ08**	7,862,581	Nzime Village
**BIZ09**	4,541,933	Nzime Village
**BIZ10**	5,891,675	Nzime Village
**BIZ11**	3,018,536	Nzime Village
**BIZ12**	6,929,884	Nzime Village
**BIZ13**	4,107,945	Nzime Village
**BIZ14**	6,975,320	Nzime Village
**BIZ15**	8,240,105	Nzime Village
**BIZ16**	9,567,489	Nzime Village
**BIZ17**	11,150,958	Nzime Village
**BIZ18**	10,350,935	Nzime Village
**BOS01**	10,973,160	Baka forest
**BOS02**	7,782,534	Baka settled
**BOS03**	9,232,735	Baka settled
**BOS04**	6,973,072	Baka settled
**BOS05**	8,737,842	Baka settled
**BOS06**	11,888,524	Baka settled
**BOS07**	9,826,837	Baka settled
**BOS08**	9,731,891	Baka settled
**BOS09**	7,191,427	Baka settled
**BOS10**	8,041,685	Baka settled
**BOS11**	9,168,116	Baka settled
**BOS12**	7,231,283	Baka forest
**BOS13**	9,816,040	Baka forest
**BOS14**	9,352,911	Baka forest
**BOS15**	8,629,551	Baka forest
**BOS16**	8,737,144	Baka forest
**BOS17**	7,711,586	Baka forest
**BOS18**	7,862,951	Baka forest
**BOS19**	7,484,896	Baka forest
**BOS20**	6,518,104	Baka forest
**BOS21**	8,716,612	Baka forest
**BOS22**	7,795,313	Baka forest
**BOS23**	4,265,113	Baka forest
**BOS24**	6,187,621	Baka forest
**BOS25**	9,443,330	Baka forest
**BOS26**	9,918,767	Baka forest

Table S3. Related to Figure 3. wpSGB list and details.

SGBs ID	Taxonomy	kSGBs in Pasolli et al 2019
BIZ06_bin.19	d__Bacteria;p__Firmicutes_A;c__Clostridia;o__Christensenellales;f__CAG-138;g__PeH17;s__PeH17 sp000435055	yes
BIZ06_bin.2	d__Bacteria;p__Firmicutes_A;c__Clostridia;o__Christensenellales;f__CAG-138;g__Phil1;s__Phil1 sp001940855	yes
BIZ07_bin.63	d__Bacteria;p__Proteobacteria;c__Gammaproteobacteria;o__Enterobacterales;f__Enterobacteriaceae;g__Escherichia;s__Escherichia coli	yes
BIZ08_bin.21	d__Bacteria;p__Campylobacterota;c__Campylobacteria;o__Campylobacterales;f__Campylobacteraceae;g__Campylobacter_D;s__Campylobacter_D sp900539255	yes
BIZ09_bin.14	d__Bacteria;p__Bacteroidota;c__Bacteroidia;o__Bacteroidales;f__Bacteroidaceae;g__Prevotella;s__Prevotella sp002265625	yes
BIZ11_bin.14	d__Bacteria;p__Actinobacteriota;c__Coriobacteriia;o__Coriobacteriales;f__Coriobacteriaceae;g__Collinsella;s__	no
BIZ11_bin.5	d__Bacteria;p__Proteobacteria;c__Gammaproteobacteria;o__Burkholderiales;f__Burkholderiaceae;g__Duodenibacillus;s__Duodenibacillus sp900767875	yes
BIZ12_bin.23	d__Bacteria;p__Proteobacteria;c__Gammaproteobacteria;o__Burkholderiales;f__Burkholderiaceae;g__Sutterella;s__	yes
BIZ16_bin.15	d__Bacteria;p__Firmicutes_A;c__Clostridia;o__Oscillospirales;f__Ruminococcaceae;g__Ruminococcus_C;s__Ruminococcus_C sp900545285	yes
BIZ18_bin.3	d__Bacteria;p__Firmicutes_A;c__Clostridia;o__Oscillospirales;f__Oscillospiraceae;g__CAG-83;s__CAG-83 sp900545495	yes
BOS02_bin.24	d__Bacteria;p__Firmicutes;c__Bacilli;o__RFN20;f__CAG-288;g__CAG-568;s__CAG-568 sp000434395	yes
BOS02_bin.33	d__Bacteria;p__Verrucomicrobiota;c__Verrucomicrobiae;o__Opitutales;f__CAG-312;g__Merdousia;s__Merdousia sp002437405	yes
BOS03_bin.9	d__Bacteria;p__Elusimicrobiota;c__Elusimicrobia;o__Elusimicrobiales;f__Elusimicrobiaceae;g__UBA1436;s__UBA1436 sp900541355	yes
BOS05_bin.27	d__Bacteria;p__Firmicutes_A;c__Clostridia;o__Oscillospirales;f__Ruminococcaceae;g__CAG-353;s__CAG-353 sp900768995	yes
BOS05_bin.8	d__Bacteria;p__Myxococcota;c__Bradymonadia;o__UBA4248;f__UBA4248;g__UBA4248;s__	no
BOS11_bin.13	d__Bacteria;p__Proteobacteria;c__Gammaproteobacteria;o__Enterobacterales;f__Succinivibrionaceae;g__Succinivibrio;s__Succinivibrio sp000431835	yes
BOS11_bin.33	d__Bacteria;p__Firmicutes_A;c__Clostridia;o__Lachnospirales;f__Lachnospiraceae;g__Acetatifactor;s__	yes
BOS12_bin.3	d__Bacteria;p__Spirochaetota;c__Spirochaetia;o__Treponematales;f__Treponemataceae;g__Treponema_D;s__Treponema_D sp900541945	yes
BOS14_bin.1	d__Bacteria;p__Firmicutes_A;c__Clostridia;o__Oscillospirales;f__Butyricicoccaceae;g__Butyricicoccus_A;s__Butyricicoccus_A sp002395695	yes
BOS14_bin.30	d__Bacteria;p__Firmicutes_A;c__Clostridia;o__Oscillospirales;f__Oscillospiraceae;g__CAG-83;s__CAG-83 sp900549395	yes
BOS21_bin.24	d__Bacteria;p__Firmicutes_A;c__Clostridia;o__Oscillospirales;f__Oscillospiraceae;g__CAG-83;s__CAG-83 sp000435975	yes
BOS22_bin.1	d__Bacteria;p__Spirochaetota;c__Spirochaetia;o__Sphaerochaetales;f__Sphaerochaetaceae;g__UBA9732;s__UBA9732 sp001940825	yes
BOS24_bin.1	d__Bacteria;p__Firmicutes_C;c__Negativicutes;o__Veillonellales;f__Dialisteraceae;g__Dialister;s__	yes
BOS24_bin.22	d__Bacteria;p__Firmicutes_A;c__Clostridia;o__Oscillospirales;f__Oscillospiraceae;g__Faecousia;s__Faecousia sp000434635	yes
BOS25_bin.44	d__Bacteria;p__Verrucomicrobiota;c__Kiritimatiellae;o__RFP12;f__UBA1067;g__RUG572;s__RUG572 sp900547945	yes
BOS26_bin.19	d__Bacteria;p__Firmicutes_C;c__Negativicutes;o__Acidaminococcales;f__Acidaminococcaceae;g__Phascolarctobacterium_A;s__Phascolarctobacterium_A sp900551335	yes

Table S6. Related to Figure 2. Age, gender and weight for the recruited subjects.

ID	age (y)	gender	weight (kg)
BOS02	28	F	NA
BOS03	30	M	NA
BOS04	30	F	NA
BOS05	47	F	NA
BOS06	44	M	NA
BOS07	32	F	NA
BOS08	61	M	45.3
BOS09	49	F	NA
BOS10	20	F	NA
BOS11	22	F	NA
BOS01	60	M	44.9
BOS12	55	F	NA
BOS13	66	F	NA
BOS14	30	M	48.9
BOS15	33	M	41.2
BOS16	66	F	37.9
BOS17	52	F	39.9
BOS18	55	F	43.8
BOS19	25	F	NA
BOS20	29	M	NA
BOS21	35	M	NA
BOS22	48	F	39
BOS23	49	F	44.4
BOS24	65	F	37.5
BOS25	34	F	NA
BOS26	48	F	35.7
BIZ04	56	F	NA
BIZ05	34	M	NA
BIZ06	53	M	NA
BIZ07	50	F	NA
BIZ08	51	M	NA
BIZ09	48	M	NA
BIZ10	54	F	NA
BIZ11	29	M	NA
BIZ12	46	M	NA
BIZ13	53	F	NA
BIZ14	63	F	NA
BIZ15	46	M	NA
BIZ16	40	F	NA
BIZ17	18	F	NA
BIZ18	39	F	NA
BIZ02	47	F	69.1
BIZ03	45	M	64.1
BIZ01	53	M	NA

Table S7. Related to Figure 2-5. Control for possible contamination: characterization of a control sample (CTRL) containing all analytical reagents from extraction to library preparation using 16S rRNA sequencing. Number of reads and taxonomy.

OTU ID	CTRL	taxonomy
4598d381ec2e1e2419695d8c29f2a708	47	k__Bacteria; p__Proteobacteria; c__Gammaproteobacteria; o__Aeromonadales; f__Aeromonadaceae
cd88b26ba20d4686293629d5d7572c14	32	k__Bacteria; p__Firmicutes; c__Clostridia; o__Clostridiales; f__Lachnospiraceae; g__; s__
7b220b76eaeb321867b0aa4315807e82	9	k__Bacteria; p__Firmicutes; c__Clostridia; o__Clostridiales; f__Ruminococcaceae; g__; s__
8dd90e2194e6421ded926f27545797f4	6	k__Bacteria; p__Bacteroidetes; c__Bacteroidia; o__Bacteroidales; f__Porphyromonadaceae; g__Parabacteroides; s__
322117051a847a704c0812f912a8c089	5	k__Bacteria; p__Actinobacteria; c__Coriobacteriia; o__Coriobacteriales; f__Coriobacteriaceae; g__Slackia; s__
